# Author Correction: Reaction-based fluorogenic probes for detecting protein cysteine oxidation in living cells

**DOI:** 10.1038/s41467-022-34953-8

**Published:** 2022-11-25

**Authors:** Renan B. Ferreira, Ling Fu, Youngeun Jung, Jing Yang, Kate S. Carroll

**Affiliations:** 1 Department of Chemistry, UF Scripps Biomedical Research, Jupiter, FL 33458 US; 2 State Key Laboratory of Proteomics, Beijing Proteome Research Center, National Center for Protein Sciences Beijing, Beijing Institute of Lifeomics, Beijing, 102206 China; 3 Innovation Institute of Medical School, Medical College, Qingdao University, Qingdao, 266071 China

**Keywords:** Fluorescent probes, Post-translational modifications, Fluorescent dyes, Sensors and probes, Kinases

Correction to: *Nature Communications* 10.1038/s41467-022-33124-z, published online 21 September 2022

The original version of this Article incorrectly omits Supplementary Data files 1–3. Additionally, references in the main Article to Supplementary Data 1 through to Supplementary Data 3 were incorrectly cited as Supplementary Table 2 through to Supplementary Table 4, respectively. This has been corrected in both the PDF and HTML versions of the Article, and the Supplementary Data files 1 through to 3 can now be found as Supplementary Information associated with this Correction.

## Supplementary information

Description of Supplementary Files

Supplementary Data 1

Supplementary Data 2

Supplementary Data 3

