# Supplementary material for: Reaction-based fluorogenic probes for detecting protein cysteine oxidation in living cells
Source: Nat Commun. 2022 Sep 21;13:5522. doi: 10.1038/s41467-022-33124-z (PMC9492777; doi:10.1038/s41467-022-33124-z)
Supplement: Supplementary file 1 — Supplementary Information [file 41467_2022_33124_MOESM1_ESM.pdf]

SUPPLEMENTARY INFORMATION

Reaction-based fluorogenic probes for detecting protein cysteine oxidation in living cells

Renan B. Ferreira[1], Ling Fu[2,3], Youngeun Jung[1], Jing Yang[2], and Kate S. Carroll[1]*
[1]Department of Chemistry, UF Scripps Biomedical Research, Jupiter, Florida 33458, United States
[2]State Key Laboratory of Proteomics, Beijing Proteome Research Center, National Center for Protein Sciences • Beijing, Beijing Institute of Lifeomics, Beijing 102206, China
[3]Innovation Institute of Medical School, Medical College, Qingdao University, Qingdao 266071, China
*e-mail: kate.carroll@ufl.edu

CONTENT	PAGE
TABLE OF CONTENTS	S1
CHEMICAL SYNTHESIS	S2–S9
TABLE S1. FLUORESCENCE PROPERTIES OF CYSOX PROBES	S10
FIG. S1. FLUORESCENT AND FLUOROGENIC PROBES FOR CYSTEINE SULFENIC ACID	S11
FIG. S2. KINETIC ANALYSIS CSA REACTION WITH COMPOUNDS **4–8**	S12
FIG. S3. FLUORESCENCE SCAN OF COMPOUNDS **4–6** AND CSA ADDUCTS	S13
FIG. S4. UV-VIS ABSORPTION AND FLUORESCENCE SPECTRA OF **7** AND CSA-**7**	S14
FIG. S5. UV-VIS ABSORPTION AND FLUORESCENCE SPECTRA OF **8** AND CSA-**8**	S15
FIG. S6. GSH DOES NOT REACT WITH CYSOX PROBES	S16
FIG. S7. C36S GPX3 DOES NOT REACT WITH CYSOX PROBES	S17
FIG. S8. CYSOX PROBES DETECT *S*-SULFENATION IN WILD-TYPE, BUT NOT C797S, EGFR	S18
FIG. S9. NO-WASH LIVE-CELL MICROSCOPY OF SULFENIC ACID IN CELLS WITH CYSOX1	S19
FIG. S10. COLOCALIZATION ANALYSIS OF CYSOX1 WITH ORGANELLES	S20
FIG. S11. HELA CELL VIABILITY ASSAY	S21
FIG. S12. FLUORESCENCE CELL IMAGING OF HELA CELLS TREATED WITH **6**, **7 OR 8**	S22
FIG. S13. *IN SITU* CYSOX2 TREATMENT DOES NOT PERTURB THE CYSTEINOME	S23
FIG. S14. PARAMETER OPTIMIZATION FOR 96-WELL ASSAY	S24
FIG. S15. SUMMARY OF GSK3 INHIBITOR POTENCY AND SELECTIVITY	S25
FIG. S16. Β-CATENIN PHOSPHORYLATION AND GLYCOGEN SYNTHASE ACTIVITY	S26
FIG. S17. IN-GEL DETECTION OF SULFENIC ACID AFTER GSK3 INHIBITOR TREATMENT	S27
FIG. S18. REPRESENTATIVE EXTRACTED ION CHROMATOGRAMS OF PRDX6 AND GAPDH	S28
NMR SPECTRA	S29–S41
SUPPLEMENTARY REFERENCES	S42

SUPPLEMENTARY METHODS

CHEMICAL SYNTHESIS

General methods: Thin layer chromatography (TLC) was performed on aluminum backed SiO_2-60 F254 TLC plates with visualization via UV light. Flash column chromatography was performed using SiO_2-60 230−400 mesh silica gel and mobile phases as indicated within procedures. Liquid chromatography-mass spectrometry (LC-MS) analyses were performed using an Agilent Technologies 1220 Infinity LC coupled to a 6120 Quadrupole LC-MS analyzer using electron spray ionization (ESI) in both negative and positive modes. ^{1}H Nuclear Magnetic Resonance (NMR), ^{13}C NMR, and ^{19}F NMR were recorded on spectrometers operating at 400 MHz for ^{1}H, 377 MHz for ^{19}F, and at 100 MHz for ^{13}C, unless otherwise indicated within characterization data. Chemical shifts (δ) are given in parts per million (ppm) relative to residual protonated solvent (DMSO-*d*$_6$: δ_H 2.50 ppm, δ_C 39.50 ppm; $CDCl_3$: δ_H 7.24 ppm, δ_C 77.0 ppm). Abbreviations used are s (singlet), d (doublet), t (triplet), q (quartet), p (pentet), m (multiplet), and bs (broad singlet). Compounds **1 – 3** were prepared according to literature procedures[1,2]. In brief, 1-napthalenethiol (1 eq) and triphosgene (3 eq) in dry DCM was added dropwise pyridine (4 eq) at 0 °C and the mixture was stirred for 4 h. After purification by column chromatography on silica gel (hexanes as eluent) the acylated intermediate (1 eq) and $AlCl_3$ (3 eq) in DCM was stirred at room temperature for 7 h. The resulting material was purified by column chromatography on silica gel (10% EtOAc in hexanes) and used in the synthesis of **13** and **14**.

<u>Synthesis of intermediates **13** and **14**</u>

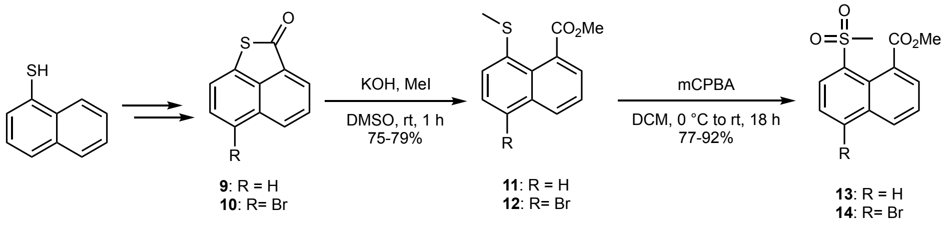

Methyl 8-(methylthio)-1-naphthoate (11): Powdered KOH (1.81 g, 32.2 mmol) was stirred in DMSO (7.1 mL) and after 5 min thiolactone[3] **9** (500 mg, 2.69 mmol) was added followed by MeI (0.67 mL, 10.7

mmol). The resulting dark green mixture gradually lightened to a yellow color and the reaction was complete after 40 min. The mixture was poured onto water (71 mL) and extracted with DCM (3 x 100 mL). The organic extracts were washed with water (50 mL) and brine (50 mL), dried ($MgSO_4$) and evaporated to yield a yellow oil. The residue was purified by column chromatography (SiO_2, 5-10% EtOAc in hexanes) to afford the title compound (474 mg, 2.04 mmol, 76% yield) as a brown oil. ^{1}H NMR ($CDCl_3$, 400 MHz): 2.44 (s, 3H), 3.97 (s, 3H), 7.45-7.51 (m, 2H), 7.62 (d, *J* = 8.0 Hz, 1H), 7.78 (t, *J* = 8.0 Hz, 2H), 7.92 (d, *J* = 8.0 Hz, 1H) ppm; ^{13}C NMR ($CDCl_3$, 100 MHz): 21.6, 52.8, 125.0, 126.4, 127.8, 128.4, 130.6, 131.4, 131.6, 132.8, 134.6, 134.7, 171.9 ppm.

Methyl 5-bromo-8-(methylthio)-1-naphthoate (12): The same procedure for the synthesis of **11** was performed with **10** as starting material, affording **12** (79% yield) as a brown oil. ^{1}H NMR ($CDCl_3$, 400 MHz): 2.42 (s, 3H), 3.96 (s, 3H), 7.57-7.63 (m, 2H), 7.68 (d, *J* = 8.0 Hz, 1H), 7.79 (d, *J* = 8.0 Hz, 1H), 8.41 (d, *J* = 8.0 Hz, 1H) ppm; ^{13}C NMR ($CDCl_3$, 100 MHz): 21.9, 53.0, 123.3, 126.6, 128.8, 130.6, 130.7, 132.0, 132.4, 132.9, 133.1, 135.1, 171.4 ppm; HRMS (*m/z*): $[M+H]^+$ calcd. for $C_{13}H_{12}BrO_2S$, 310.9736; found, 310.9743 (error: 2.15 ppm).

Methyl 8-(methylsulfonyl)-1-naphthoate (13): To a stirred solution of **11** (100 mg, 0.430 mmol) in DCM (21 mL) was added mCPBA (0.241 g, 2.15 mmol) in batches at 0 ^{o}C. The resulting reaction mixture was allowed to stir at rt with monitoring. After the completion (18 h), the reaction mixture was quenched by the addition of small amount of DMSO. Organic phase was washed with water (10 mL), saturated sodium bicarbonate (2 x 10 mL) and brine (10 mL), dried over anhydrous magnesium sulfate, filtered, and evaporated to give the crude product, which was then purified by silica gel column chromatography (SiO_2, 20-40% EtOAc in hexanes) to afford the product (91.7 mg, 0.347 mmol, 81% yield) as a white solid. ^{1}H NMR ($CDCl_3$, 400 MHz): 3.49 (s, 3H), 4.01 (s, 3H), 7.57 (t, *J* = 7.2 Hz, 1H), 7.66 (t, *J* = 8.0 Hz, 1H), 7.96 (d, *J* = 8.0 Hz, 1H), 8.02 (d, *J* = 8.0 Hz, 1H), 8.12 (d, *J* = 8.0 Hz, 1H), 8.42 (d, *J* = 8.0 Hz, 1H) ppm; ^{13}C NMR ($CDCl_3$, 100 MHz): 44.2, 52.7, 125.3, 125.6, 125.7, 130.1, 130.6, 132.6, 133.5, 134.8, 135.5, 138.1, 170.2 ppm.

Methyl 5-bromo-8-(methylsulfonyl)-1-naphthoate (14): The same procedure for the synthesis of **13** was performed with **12** as starting material, affording **14** (77% yield) as a white solid. ^{1}H NMR ($CDCl_3$, 400 MHz): 3.48 (s, 3H), 4.01 (s, 3H), 7.67 (t, *J* = 7.2 Hz, 1H), 7.98-8.06 (m, 2H), 8.22 (d, *J* = 8.0 Hz, 1H), 8.55 (d, *J* = 8.0 Hz, 1H) ppm; ^{13}C NMR ($CDCl_3$, 100 MHz): 44.5, 52.9, 127.1, 127.2, 130.0, 130.7, 131.2, 131.4, 131.9, 133.2, 133.3, 138.4, 169.7 ppm; HRMS (*m/z*): $[M+H]^+$ calcd. for $C_{13}H_{12}BrO_4S$, 344.9634; found, 344.9620 (error: 1.95 ppm).

<u>Synthesis of **4** and **5**</u>

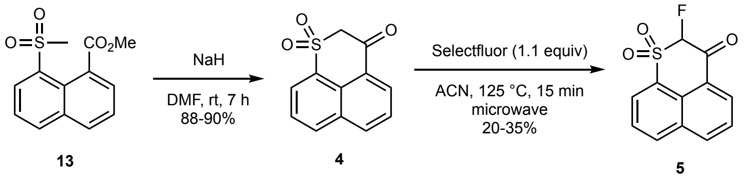

Benzo[de]thiochromen-3(2H)-one 1,1-dioxide (4): To a stirred solution of NaH (60% in mineral oil, 150 mg, 3.75 mmol, 2 equiv) in dry DMF (1.3 mL) was added dropwise a solution of the **13** (495 mg, 1.87 mmol) in DMF (2.5 mL). Resulting reaction mixture was allowed to stir under nitrogen with monitoring. After the completion (7 h), the reaction mixture was acidified to pH 3 with 2N HCl. Aqueous layer was extracted with ethyl acetate (5 x 13 ml). Combined organic layers were washed with water (2 x 25 ml) and brine (1 x 25 ml). Organic layer was then dried over anhydrous magnesium sulfate, filtered, and evaporated to dryness. Crude liquid was purified by column chromatography (SiO_2, 0-3% MeOH in DCM) to obtain the product (381 mg, 1.64 mmol, 88% yield) as an off-white solid. ^{1}H NMR ($CDCl_3$, 400 MHz): 4.58 (s, 2H), 7.77-7.84 (m, 2H), 8.27 (t, *J* = 8.0 Hz, 2H), 8.41 (d, *J* = 8.0 Hz, 1H), 8.50 (d, *J* = 8.0 Hz, 1H) ppm; ^{13}C NMR ($CDCl_3$, 100 MHz): 63.4, 125.6, 126.2, 126.8, 127.4, 128.0, 130.6, 133.4, 134.2, 134.7, 135.6, 184.8 ppm; HRMS (*m/z*): $[M+H]^+$ calcd. for $C_{12}H_9O_3S$, 233.0267; found, 233.0274 (error: 3.00 ppm).

2-Fluorobenzo[de]thiochromen-3(2H)-one 1,1-dioxide (5): Into a septum-sealed microwave tube, **4** (50.0 mg, 0.215 mmol), ACN (2 mL) and selectfluor (76.3 mg, 0.215 mmol) were charged. The resulting mixture was irradiated in a microwave cavity at 125 °C (by modulation of power). The reaction mixture

was partitioned between EtOAc and water and the aqueous phase was extracted with EtOAc (3x). The combined organic layers were dried over $MgSO_4$, filtered, and concentrated. The obtained crude compound was purified by column chromatography (0-3% MeOH in DCM) to afford the product **5** (14.2 mg, 0.0567 mmol, 26% yield) as a pale brown solid. ^{1}H NMR ($CDCl_3$, 400 MHz): 5.95 (d, J = 48.0 Hz, 1H), 7.80-7.88 (m, 2H), 8.31 (d, J = 8.0 Hz, 2H), 8.43-8.51 (m, 2H) ppm; ^{13}C NMR ($CDCl_3$, 100 MHz): 98.0 (d, $^1J_{C\text{-}F}$ = 229 Hz), 126.2, 126.5, 127.6, 127.7, 131.4, 131.5, 131.5, 133.1, 135.4, 135.9, 183.5 (d, J = 17 Hz) ppm; HRMS (*m/z*): $[M+H]^+$ calcd. for $C_{12}H_8FO_3S$, 251.0173; found, 251.0180 (error: 2.71 ppm).

<u>Synthesis of **7** (CysOx1)</u>

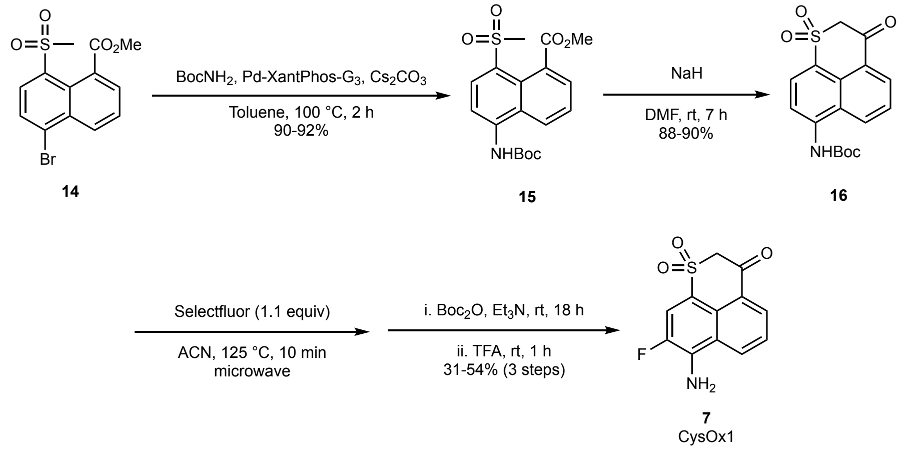

Methyl 5-((tert-butoxycarbonyl)amino)-8-(methylsulfonyl)-1-naphthoate (15): The brominated sulfone **14** (85.8 mg, 0.250 mmol), O-*t*-butyl-carbamate (35.2 mg, 0.300 mmol, 1.2 equiv.), G3-xantphos (2.4 mg, 0.0025 mmol, 0.01 equiv.), Cs_2CO_3 (122 mg, 0.375 mmol, 1.5 equiv.) in toluene (2.5 mL) were allowed to react at 100 °C for 1.5 h. At the completion of the reaction, H2O (ca. 15 mL) was added, and the mixture was extracted with DCM (3 x 30 mL). The organic phases combined were dried over MgSO4, filtered and concentrated. The crude product was purified by column chromatography (SiO_2, 20-40% EtOAc in hexanes) to afford the product (86.8 mg, 0.229 mmol, 92% yield) as an off-white solid. ^{1}H NMR ($CDCl_3$, 400 MHz): 3.43 (s, 3H), 4.00 (s, 3H), 7.20 (bs, 1H), 7.57 (dd, J = 8.0 Hz, 1H), 7.96 (d, J = 8.0 Hz, 1H), 8.05 (d, J = 8.0 Hz, 1H), 8.24 (d, J = 8.0 Hz, 1H), 8.36 (d, J = 8.0 Hz, 1H) ppm; ^{13}C NMR ($CDCl_3$, 100 MHz): 28.3, 44.4, 52.7, 82.1, 115.8, 124.0, 125.5, 126.2, 126.7, 130.4, 130.7, 132.5, 134.2, 139.3,

152.3, 169.9 ppm; HRMS (*m/z*): $[M+H]^+$ calcd. for $C_{18}H_{22}NO_6S$, 380.1162; found, 380.1172 (error: 2.42 ppm).

Tert-butyl (1,1-dioxido-3-oxo-2,3-dihydrobenzo[de]thiochromen-7-yl)carbamate (16): To a stirred solution of NaH (60% in mineral oil, 27.5 mg, 0.687 mmol, 3 equiv) in dry DMF (1.5 mL) was added dropwise a solution of **15** (86.8 mg, 0.229 mmol) in DMF (2.5 mL). Resulting reaction mixture was allowed to stir under nitrogen with monitoring. After the completion (7 h), the reaction mixture was acidified to pH 3 with 2N HCl. Aqueous layer was extracted with ethyl acetate (5 x 20 ml). Combined organic layers were washed with water (2 x 40 ml) and brine (1 x 40 ml). Organic layer was then dried over anhydrous magnesium sulfate, filtered, and evaporated to dryness. Crude liquid was purified by column chromatography (SiO_2, 0-3% MeOH in DCM) to obtain the product (73.4 mg, 0.211 mmol, 92% yield) as a yellow solid. ^1H NMR ($CDCl_3$, 400 MHz): 1.60 (s, 9H), 4.54 (s, 2H), 7.33 (bs, 1H), 7.74 (dd, *J* = 8.0 Hz, 1H), 8.22 (d, *J* = 8.0 Hz, 1H), 8.30-8.38 (m, 2H), 8.45 (d, *J* = 8.0 Hz, 1H) ppm; ^{13}C NMR ($CDCl_3$, 100 MHz): 28.3, 63.2, 82.4, 115.5, 124.7, 126.6, 126.7, 127.3, 127.7, 127.8, 128.4, 130.3, 139.6, 152.2, 184.9 ppm; HRMS (*m/z*): $[M+H]^+$ calcd. for $C_{17}H_{18}NO_5S$, 348.0900; found, 348.0909 (error: 2.52 ppm).

7-Amino-8-fluorobenzo[de]thiochromen-3(2H)-one 1,1-dioxide (7 or CysOX1): Into a septum-sealed microwave tube, the substrate **16** (139 mg, 0.400 mmol), ACN (14 mL) and Selectfluor (142 mg, 0.400 mmol) were charged. The resulting mixture was irradiated in a microwave cavity at 125 ^oC (by modulation of power) for 5 min. The mixture was diluted with water (30 mL) and extracted with DCM (3 x 50 mL). The combined organic phases were dried with MgSO4, filtered, and concentrated to afford the crude mixture of desired product and non-fluorinated by-product, which was applied to the next step of Boc protection. To an ice-cooled stirred solution of the aniline mixture (0.615 mmol) and pyridine (297 uL, 3.69 mmol, 6 equiv) in DCM (12 mL), added di-tert-butyl dicarbonate (311 μL, 1.35 mmol, 2.2 equiv) dropwise and the reaction mixture was allowed to stir at rt for 4 h. The mixture was then diluted with water (30 mL) and extracted with DCM (3 x 50 mL). The combined organic phases were dried with MgSO4, filtered and concentrated. The crude mixture was purified by column chromatography (SiO2, 1-10% EtOAc in DCM) to afford the desired fluorinated intermediate (49.7 mg, 0.136 mmol, 34% yield) and

the recovered starting material **16** (32.8 mg, 0.0944 mmol, 24% recovery). The fluorinated intermediate (0.136 mmol) was dissolved in TFA (5 mL) and stirred at rt for 1 h. The solvent was removed under vacuum, the residue was resuspended in DCM, neutralized with Et_3N (16 µL) and concentrated under reduced pressure to afford the desired product **7** (CysOx1) (quant. yield) as an orange solid. ^{1}H NMR (DMSO-d_6, 400 MHz): 5.01 (s, 2H), 7.20 (s, 2H), 7.73-7.80 (m, 1H), 8.07 (d, J = 8.0 Hz, 1H), 8.29 (d, J = 8.0 Hz, 1H), 8.75 (d, J = 8.0 Hz, 1H) ppm; ^{13}C NMR (DMSO-d_6, 125 MHz): 62.4, 113.8 (d, J = 18.8 Hz), 117.2 (d, J = 5.0 Hz), 122.3 (d, J = 5.0 Hz), 124.5 (d, J = 20.0 Hz), 127.1, 128.4, 129.3 (d, J = 5.0 Hz), 137.6 (d, J = 10.0 Hz), 143.2 (d, $^{1}J_{C\text{-}F}$ = 196.3 Hz), 186.1 ppm; HRMS (*m/z*): $[M+H]^+$ calcd. for $C_{12}H_9FNO_3S$, 266.0282; found, 266.0289 (error: 2.67 ppm).

Synthesis of **8** (CysOx2)

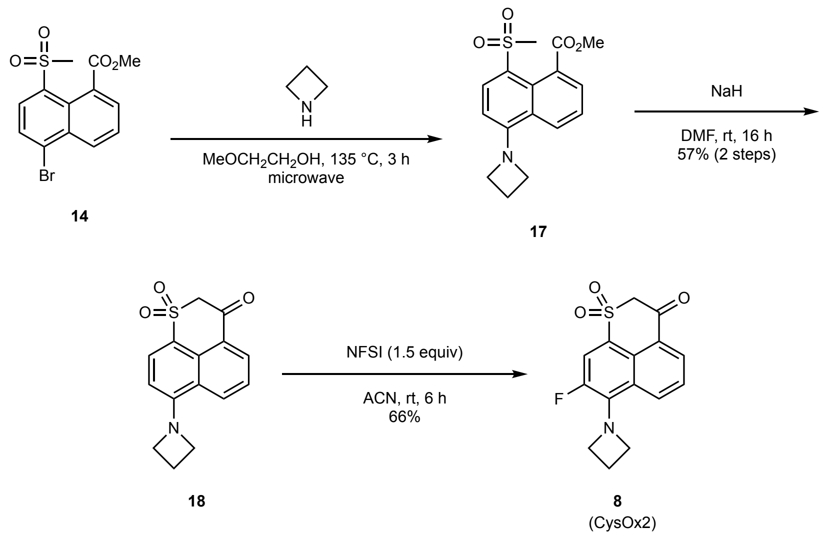

7-(Azetidin-1-yl)benzo[de]thiochromen-3(2H)-one 1,1-dioxide (18): Azetidine (426 uL, 6.33 mmol, 10 equiv) was added to a solution of the brominated starting material **14** (217 mg, 0.633 mmol) in 2-methoxyethanol (15 mL) in a microwave tube. The tube was sealed and heated to 135 °C and the reaction was monitored by TLC and LCMS. After every hour, another aliquot of azetidine (213 uL, 3.17 mmol, 5 equiv) was added and the reaction mixture was returned to the microwave reactor. At the end of 3 h, the reaction was mostly complete. The reaction mixture was then diluted in water (30 mL) and extracted with DCM (3 x 50 mL). The combined organic phases were dried over MgSO4, filtered, and

concentrated under vacuum to afford the crude azetidine derivative **17**. LCMS analysis indicated the product was pure enough that could be used in the next step without further purification. To a stirred solution of NaH (60% in mineral oil, 50.6 mg, 1.27 mmol, 2 equiv) in dry DMF (10 mL) was added dropwise a solution of the crude **17** (0.100 mmol) in DMF (15 mL). Resulting reaction mixture was allowed to stir under nitrogen with monitoring. After the completion (16 h), the reaction mixture was acidified to pH 3 with 2N HCl. Aqueous layer was extracted with ethyl acetate (5 x 30 mL). Combined organic layers were washed with water (2 x 50 mL) and brine (1 x 50 mL). Organic layer was then dried over anhydrous magnesium sulfate, filtered, and evaporated to dryness. Crude liquid was purified by column chromatography (SiO_2, 0-3% EtOAc in DCM) to obtain the product **18** (90.6 mg, 0.315 mmol, 50% yield) as a yellow solid. ^{1}H NMR ($CDCl_3$, 400 MHz): 2.56 (p, *J* = 8.0 Hz, 2H), 4.40-4.48 (m, 6H), 6.45 (d, *J* = 8.0 Hz, 1H), 7.50-7.58 (m, 1H), 8.15 (d, *J* = 8.0 Hz, 1H), 8.30 (d, *J* = 8.0 Hz, 1H), 8.49 (d, *J* = 8.0 Hz, 1H) ppm; ^{13}C NMR ($CDCl_3$, 100 MHz): 17.1, 55.7, 63.0, 105.0, 120.1, 123.2, 124.0, 127.0, 127.9, 129.6, 130.2, 131.2, 153.0, 185.9 ppm; HRMS (*m/z*): $[M+H]^+$ calcd. for $C_{15}H_{14}NO_3S$, 288.0689; found, 288.0695 (error: 2.08 ppm).

7-(Azetidin-1-yl)-8-fluorobenzo[de]thiochromen-3(2H)-one 1,1-dioxide (8 or CysOx2**):** To a solution of **18** (90.6 mg, 0.315 mmol) in ACN (15 mL), NFSI (99.4 mg, 0.315 mmol, 1 equiv) was charged. The resulting mixture was stirred and monitored by LC-MS. After 4 h, considerable amount of starting material was observed and another portion of NFSI (16.6 mg, 0.053 mmol, 0.5 equiv). After additional 2 h, the consumption of starting material stagnated again, and the reaction was quenched with the addition of water (20 mL). The mixture was extracted with DCM (3 x 40 mL). The combined organic phases were dried over $MgSO_4$, filtered, and concentrated to afford the crude material as an orange viscous oil. Purification by column chromatography (SiO_2, 0-10% EtOAc in DCM) afforded compound **8** (CysOx2) (10.7 mg, 0.0349 mmol, 33% yield) as an orange solid. ^{1}H NMR ($CDCl_3$, 600 MHz): 2.53 (p, *J* = 7.5 Hz, 2H), 4.47 (s, 2H), 4.70 (dt, *J* = 4.0 and 7.5 Hz, 4H), 7.52-7.59 (m, 1H), 7.99 (d, $^3J_{H\text{-}F}$ = 12.0 Hz, 1H) , 8.32-8.38 (m, 2H) ppm; ^{13}C NMR ($CDCl_3$, 125 MHz): 18.5 (d, *J* = 2.5 Hz), 58.7 (d, *J* = 5.0 Hz), 63.1, 105.0, 116.4 (d, *J* = 22.5 Hz), 120.0 (d, *J* = 5.0 Hz), 124.3, 125.1 (d, *J* = 5.0 Hz), 126.4, 128.0, 129.4, 130.8 (d,

J = 5.0 Hz), 140.4 (d, J = 6.3 Hz), 146.1 (d, $^1J_{C\text{-}F}$ = 201.3 Hz), 185.3 ppm; ; HRMS (m/z): $[M+H]^+$ calcd. for $C_{15}H_{13}FNO_3S$, 306.0595; found, 306.0604 (error: 3.00 ppm).

Synthesis of **6**

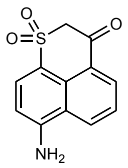

6

7-Aminobenzo[de]thiochromen-3(2H)-one 1,1-dioxide (6 or H-SNNH2**):** To a round-bottomed flask charged with **16** (100 mg, 0.288 mmol) under N2 atmosphere, TFA (10 mL) was added. The solution was stirred for 1 h. The solvent was removed under vacuum, the residue was resuspended in DCM, neutralized with Et_3N (16 µL) and concentrated under reduced pressure to afford the compound **6** (quant. yield) as an orange solid. ^{1}H NMR (DMSO-d_6, 400 MHz): 4.90 (s, 2H), 6.87 (d, J = 8.0 Hz, 1H), 7.22 (s, 2H), 7.67-7.73 (m, 1H), 8.00 (d, J = 8.0 Hz, 1H), 8.29 (d, J = 8.0 Hz, 1H), 8.65 (d, J = 8.0 Hz, 1H) ppm; ^{13}C NMR (DMSO-d_6, 100 MHz): 63.0, 105.9, 118.0, 121.2, 124.2, 127.1, 127.5, 128.5, 129.6, 130.0, 151.6, 187.1 ppm.

Preparation of probe-CSA adducts. To a solution of dipeptide cyclic sulfonamide CSA (36.6 mg, 0.100 mmol) in DCM (3.0 mL) was added a C-nucleophile probe (0.100 mmol, 1 equiv), followed by Et_3N (13.9 µL, 0.100 mmol, 1 equiv). The reaction mixture was stirred at rt overnight. LC-MS analysis of the reaction mixture indicated complete consumption of the starting materials and the formation of the desired CSA-probe adduct as a single product. The reaction mixture was neutralized with TFA, diluted in DCM (20 mL), and washed with water (20 mL) and brine (20 mL). The organic phase was dried over $MgSO_4$, filtered and concentrated. The obtained products were purified via column chromatography (SiO_2, 0-100% EtOAc in hexanes) and used as is in subsequent experiments.

Supplementary Table 1 | Fluorescence properties of probes CysOx1 or CysOx2 and their CSA adducts in PBS (pH = 7.4).

Compound	λ_{MAX} excitation (nm)	λ_{MAX} emission (nm)	Stokes shift (nm)	Extinction coefficient (10^3 M^{-1} cm^{-1})	Quantum yield (φ, %)
CysOx1	440	569	129	2.32±0.04	0.44
CSA-CysOx1	313, 443	572	129	3.88±0.03	0.93
CysOx2	394	531	137	0.71±0.06	0.91
CSA-CysOx2	335, 447	606	159	4.56±0.04	2.39

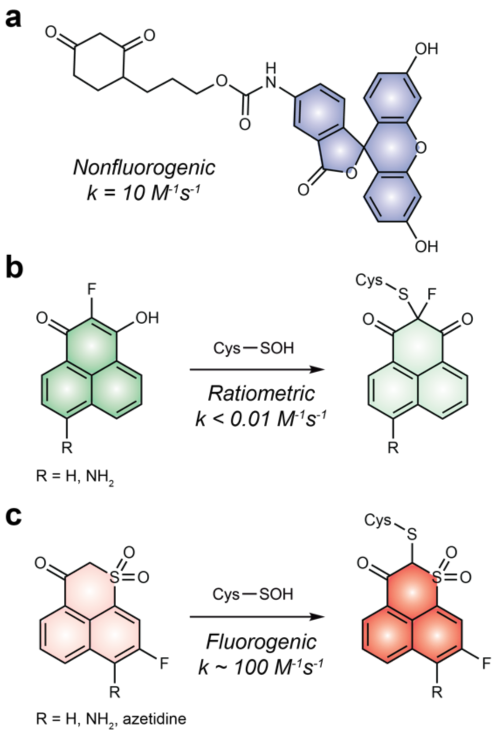

Supplementary Fig. 1 | Fluorescent (**a**,**b**) and fluorogenic (**c**) probes for cysteine sulfenic acid.

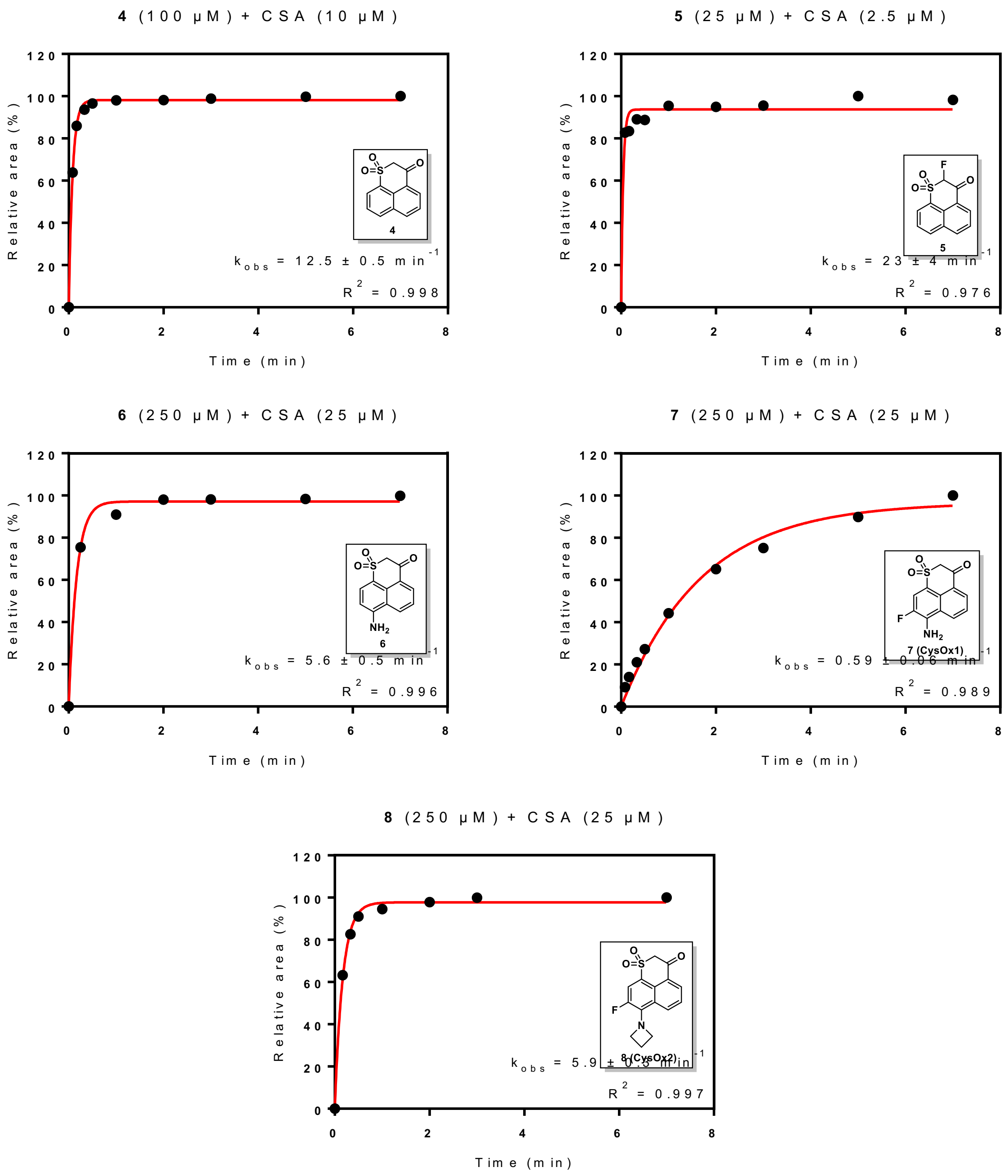

Supplementary Fig. 2 | Kinetic analysis of compounds **4**–**8** from main text Fig. 2.

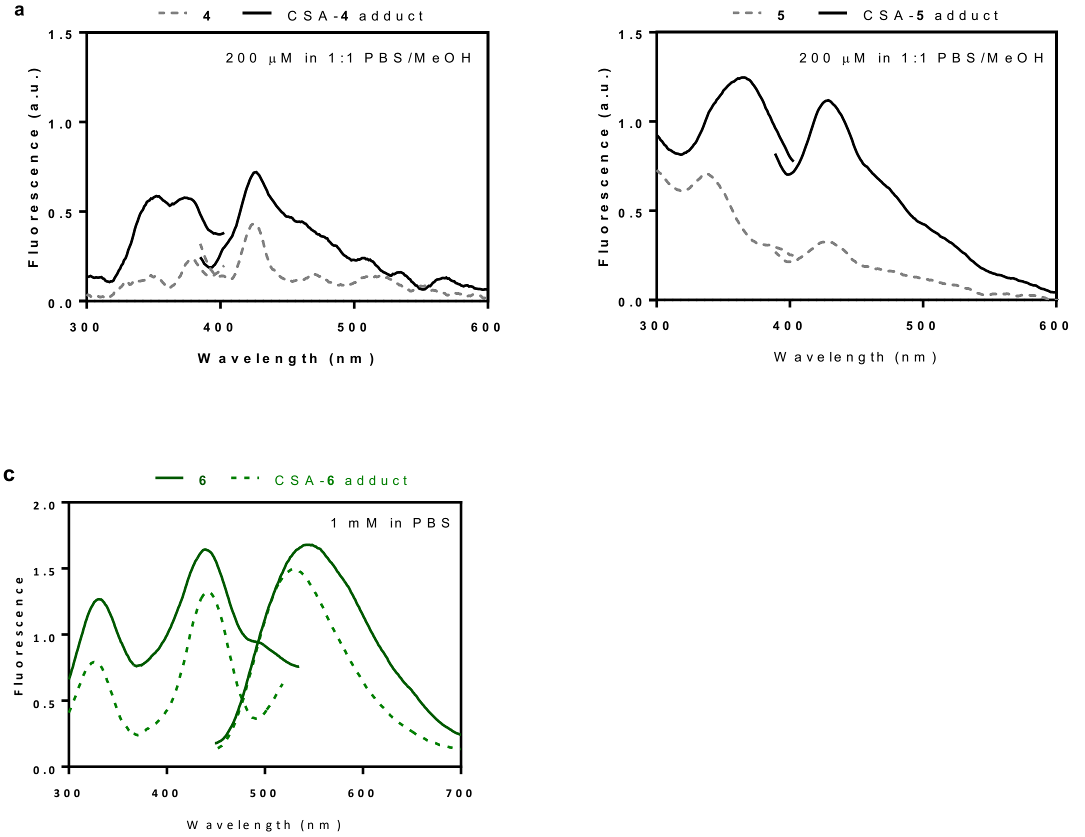

Supplementary Fig. 3 | Fluorescence scan of compounds **4**–**6** and CSA adducts. **a-b**, Emission and excitation fluorescence spectra of **4-5** and CSA adducts in 1:1 PBS/MeOH. **c**, Emission and excitation fluorescence spectra of **6** and its CSA-**6** in PBS (pH 7.4).

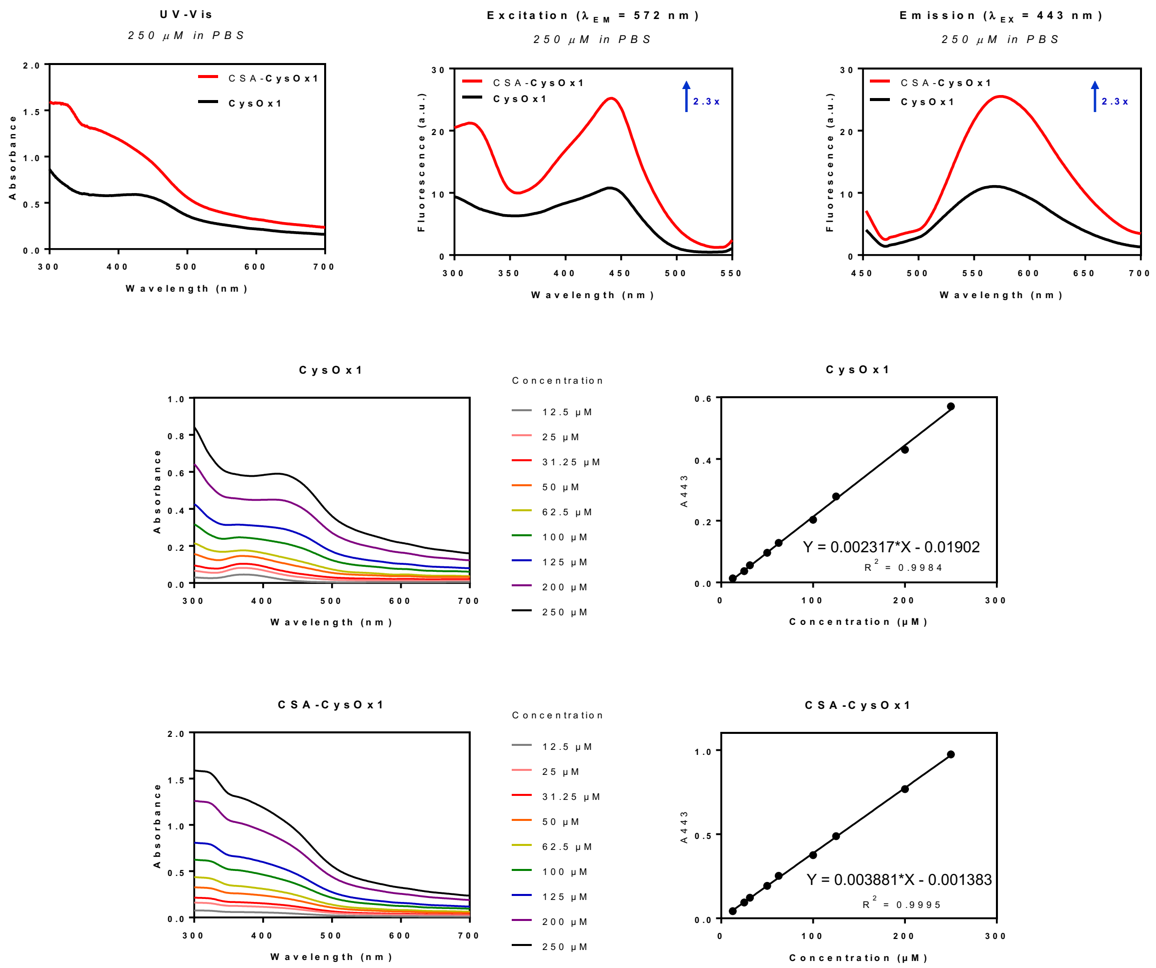

Supplementary Fig. 4 | UV-Vis absorption and fluorescence spectra of **7** (CysOx1) and CSA-**7** (CSA-CysOx1) solutions in PBS (pH 7.4).

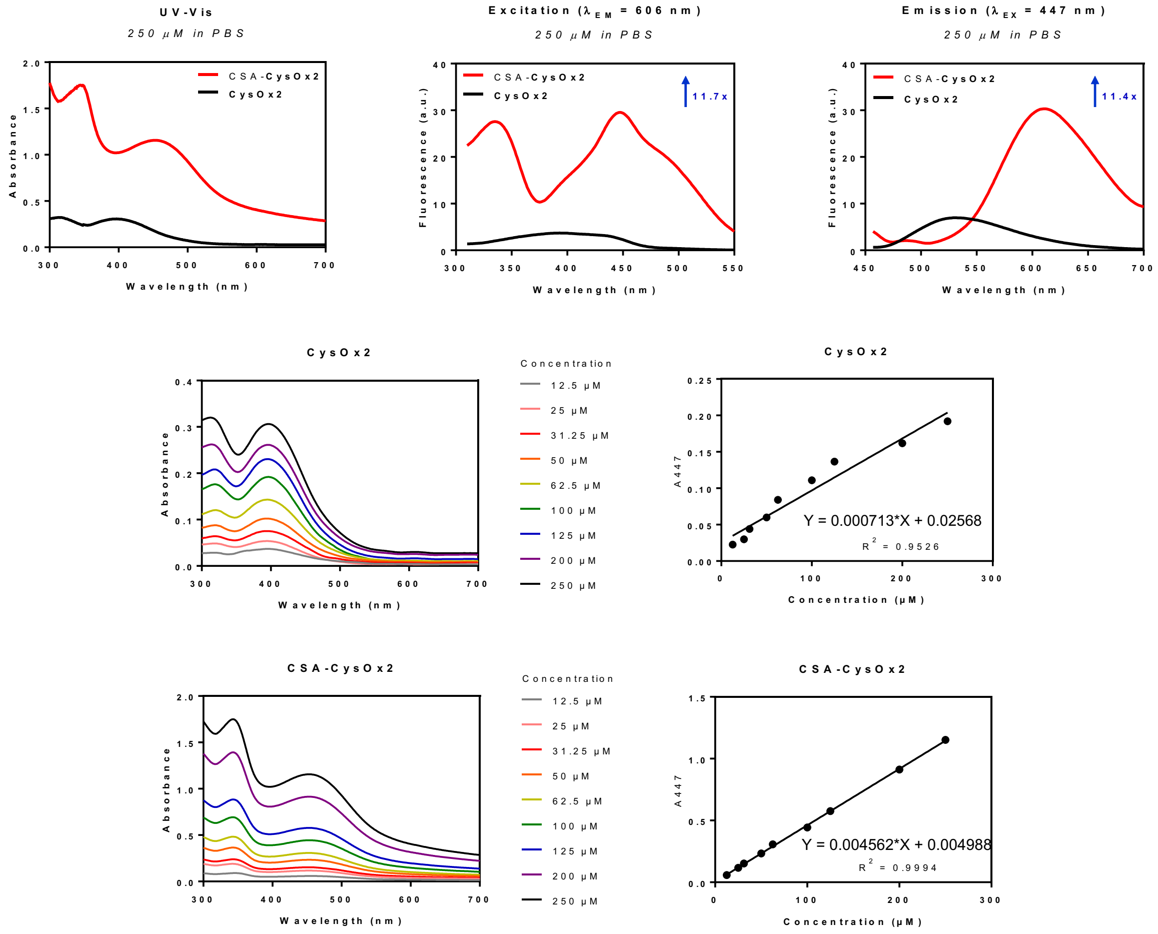

Supplementary Fig. 5 | UV-Vis absorption and fluorescence spectra of **8** (CysOx2) and CSA-**8** (CSA-CysOx2) solutions in PBS (pH 7.4).

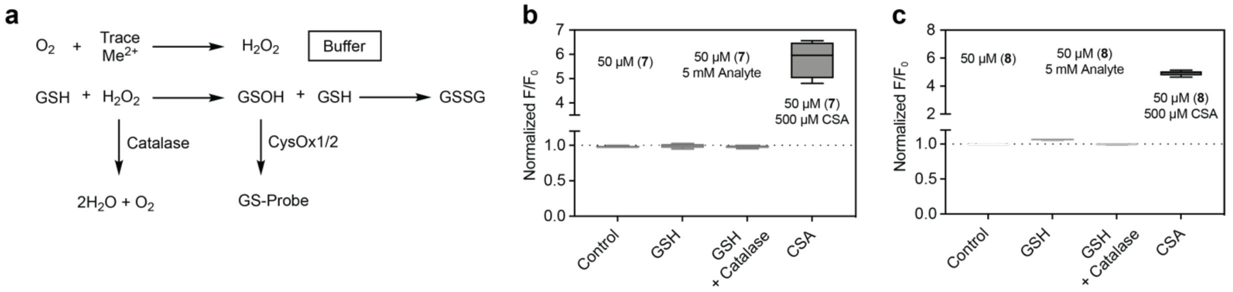

Supplementary Fig. 6 | GSH does not react with CysOx probes. **a,** Hydrogen peroxide forms in the presence of oxygen and trace metal ions present in buffer solution. H_2O_2 slowly oxidizes GSH to GSOH, which can react with **7** or **8** (CysOx1 or CysOx2) or GSH. When present, catalase metabolizes H_2O_2 and prevents GSOH formation. **b-c,** Box and whisker plot of normalized fluorescence intensity (F/F_0) for reaction between **7** or **8** (CysOx1 or CysOx2; 50 μM) and GSH (5 mM) with or without catalase (1 U) as compared to CSA positive control after 1 h. Box plots show center line as median, whiskers show maxima and minima, and box limits show upper and lower quartiles. *N* = *4* independent experiments.

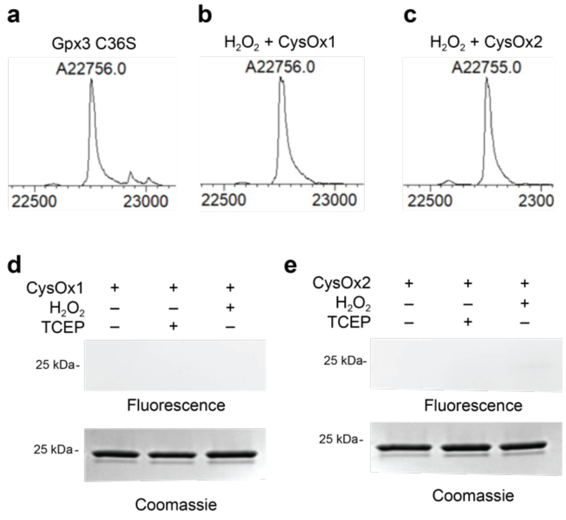

Supplementary Fig. 7 | C36S Gpx3 does not react with CysOx probes. **a-c,** Intact MS analysis of C36S Gpx3 (10 μM) and its reaction products with H_2O_2 (15 μM) and CysOx1 or CysOx2 (1 mM) after 1 h in 50 mM HEPES pH 7.4. **d-e,** In-gel fluorescence analysis of reaction products between Gpx3 (10 μM) and CysOx1 or CysOx2 (1 mM) with or without H_2O_2 (15 μM) or TCEP (1 mM) after 1 h in 50 mM HEPES pH 7.4. *N = 2* independent experiments for each probe.

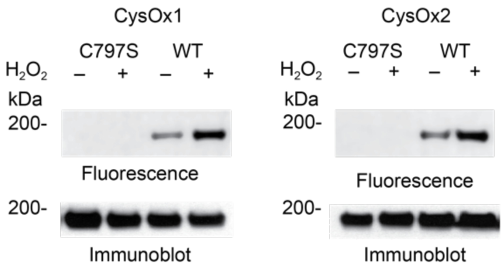

Supplementary Fig. 8 | CysOx probes detect *S*-sulfenation in wild-type, but not C797S, EGFR. HeLa cells were transfected with wild-type or C797S EGFR pCMV6-XL-4. After 24 h, cells were treated with or without H_2O2 (500 µM) and then incubated with CysOx1 or CysOx2 (50 µM) for 1 h at 37 °C. Cell lysates were prepared, EGFR immunoprecipitated, and analyzed for *S*-sulfenation (in gel fluorescence) and total EGFR (immunoblot). *N = 2* independent experiments for each probe.

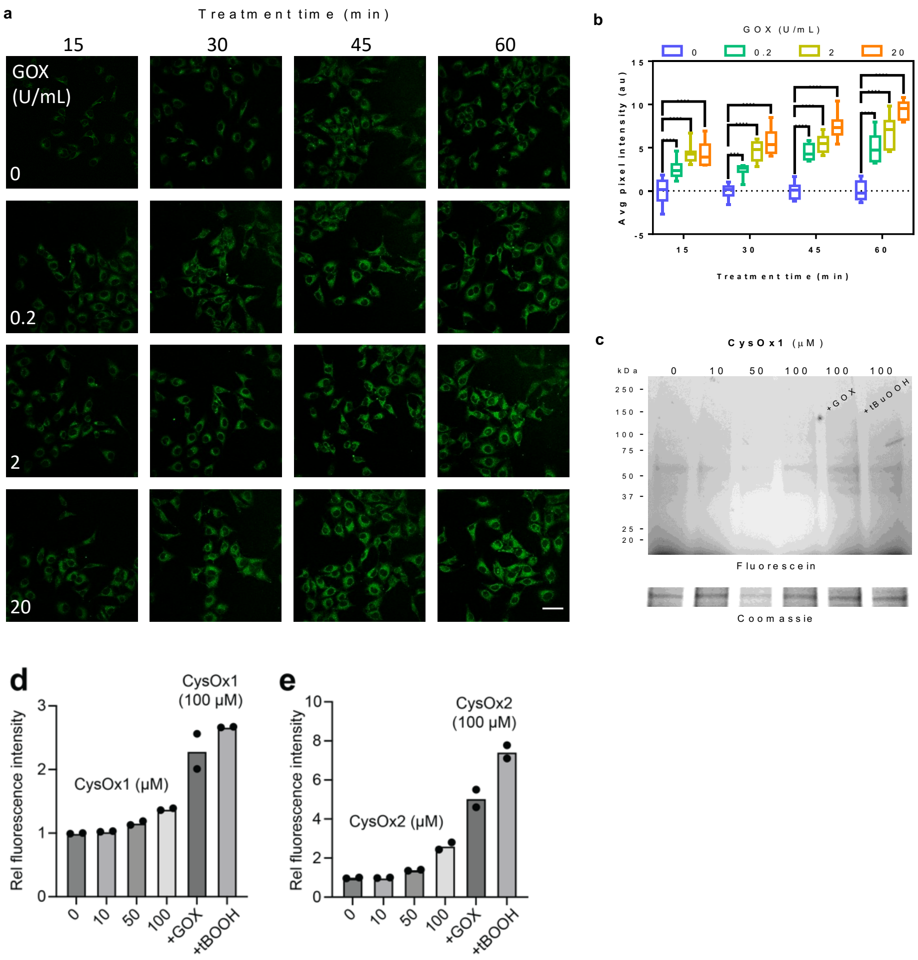

Supplementary Fig. 9 | No-wash live-cell microscopy of cysteine sulfenic acid in cells with CysOx1. **a,** No-wash live-cell confocal images of HeLa cells at different time points after the addition of (10 μM) and GOX at the indicated concentrations (0-20 U/mL). λ_{ex} = 458 nm; scale bar: 50 μM. Representative images are shown from *N = 3* independent experiments. **b,** Box and whisker plot of the average pixel intensities from panel **a**. Data are representative of *N* = 10 independent readings from 5 different frames. Error bars are ±SEM. Variance was analyzed by two-way ANOVA test. ns P > 0.05, * P < 0.05, ** P < 0.01, *** P < 0.001, **** P < 0.0001 when compared against cells treated with probe only. Box plots show center line as median, whiskers show maxima and minima, and box limits show upper and lower quartiles. **c,** In-gel fluorescence analysis of lysates derived from HeLa cells incubated with CysOx1 and GOX (20 U/mL) or *t*-BOOH (200 μM). *N* = 3 independent experiments. **d**, *S*-sulfenation was quantified as total fluorescent signal per lane and expressed relative to the control without CysOx1. **e,** Same as **d**, except with CysOx2. Data in **d-e** are representative of *N* = 2 independent experiments.

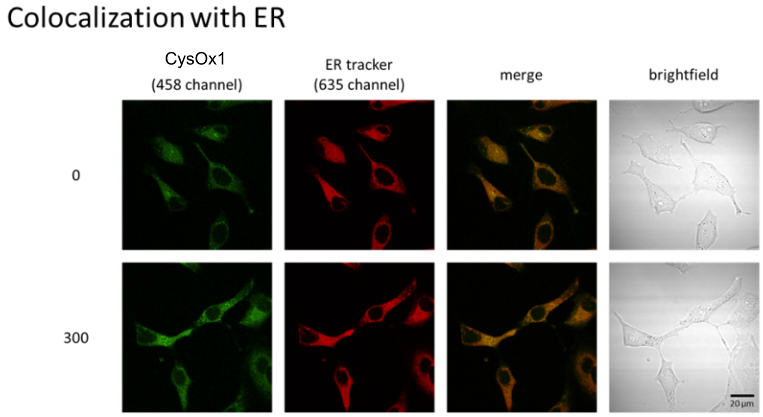

	no H_2O_2		300 µM H_2O_2	
	full image	ROI	full image	ROI
Pearson's r (-1 to 1)	0.83	0.61	0.85	0.59
Li's ICQ (-0.5 to 0.5)	0.335	0.228	0.360	0.208
Costes p-value	1.000	1.000	1.000	1.000
Ratio of randomized images with higher correlation than original image	0/100	0/100	0/100	0/100

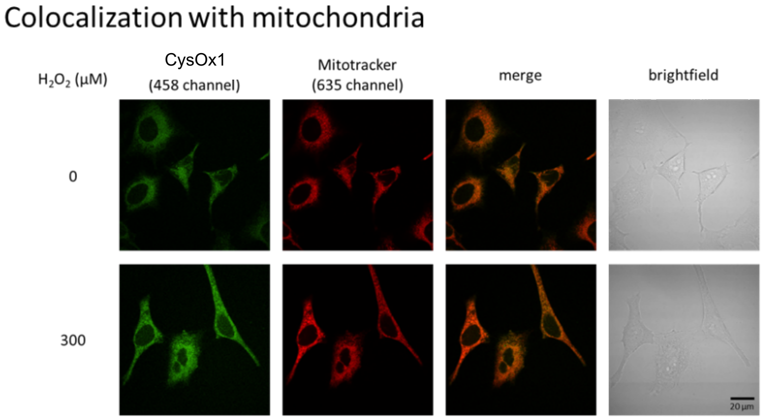

	no H_2O_2		300 µM H_2O_2	
	full image	ROI	full image	ROI
Pearson's r (-1 to 1)	0.86	0.80	0.87	0.80
Li's ICQ (-0.5 to 0.5)	0.386	0.352	0.381	0.353
Costes p-value	1.000	1.000	1.000	1.000
Ratio of randomized images with higher correlation than original image	0/100	0/100	0/100	0/100

Supplementary Fig. 10 | HeLa cell fluorescence imaging analysis of colocalization of CysOx1 (50 µM) incorporation and organelle-selective dyes for ER and mitochondria. Treatments were performed in EMEM supplemented with 0.1% DMSO for 60 min at 37 °C. The data iondicate that no preference for colocalization of probe incorporation in these organelles in the presence or absence of H_2O_2. Representative images are shown from *N* = *3* independent experiments.

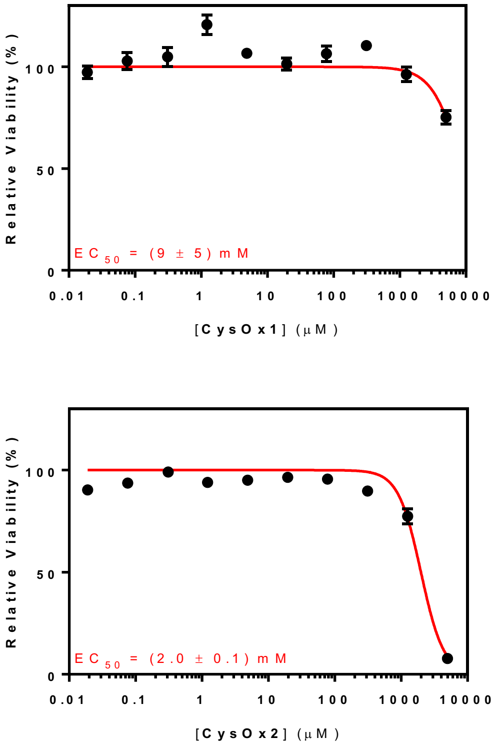

Supplementary Fig. 11 | HeLa cell viability assay. HeLa cells were treated with variable concentrations of CysOx probes in DPBS containing 1% DMSO at 37 °C for 1 h, followed by addition of the CellTiter-Glo® reagent. Data are representative of *N* = 10 independent readings per condition and were normalized to the untreated control. Error bars are ±SEM. Nonlinear fitting was performed using the equation for [inhibitor] vs. normalized response – variable slope. *N* = 3 independent experiments.

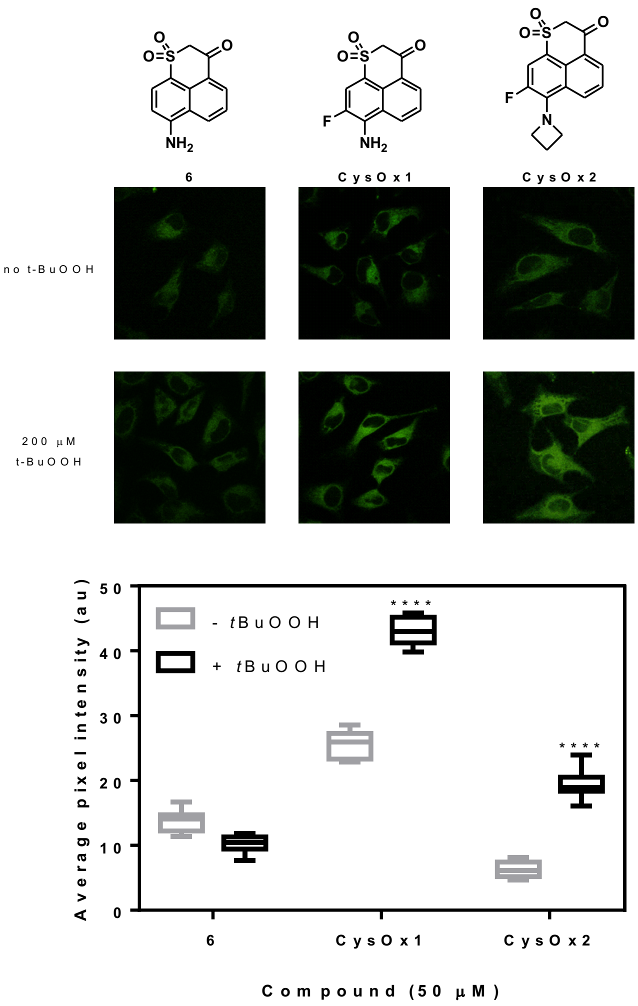

Supplementary Fig. 12 | Fluorescence cell imaging of HeLa cells treated with **6**–**8**. HeLa cells were pre-treated with PBS or t-BuOOH (200 µM) in EMEM supplemented with 0.1% DMSO for 10 min, followed by treatment with compounds **6** or CysOx1 or CysOx2 (50 µM) for 15 min. Cells were then washed and analyzed on the microscope. λ_{ex} = 458 nm; scale bar: 20 µM. Data are representative of *N* = 10 independent readings from 5 different frames. Variance was analyzed by two-way ANOVA test. ns $P > 0.05$, * $P < 0.05$, ** $P < 0.01$, *** $P < 0.001$, **** $P < 0.0001$ when compared against cells treated with probe only. Box plots show center line as median, whiskers show maxima and minima, and box limits show upper and lower quartiles. *N* = 3 independent experiments.

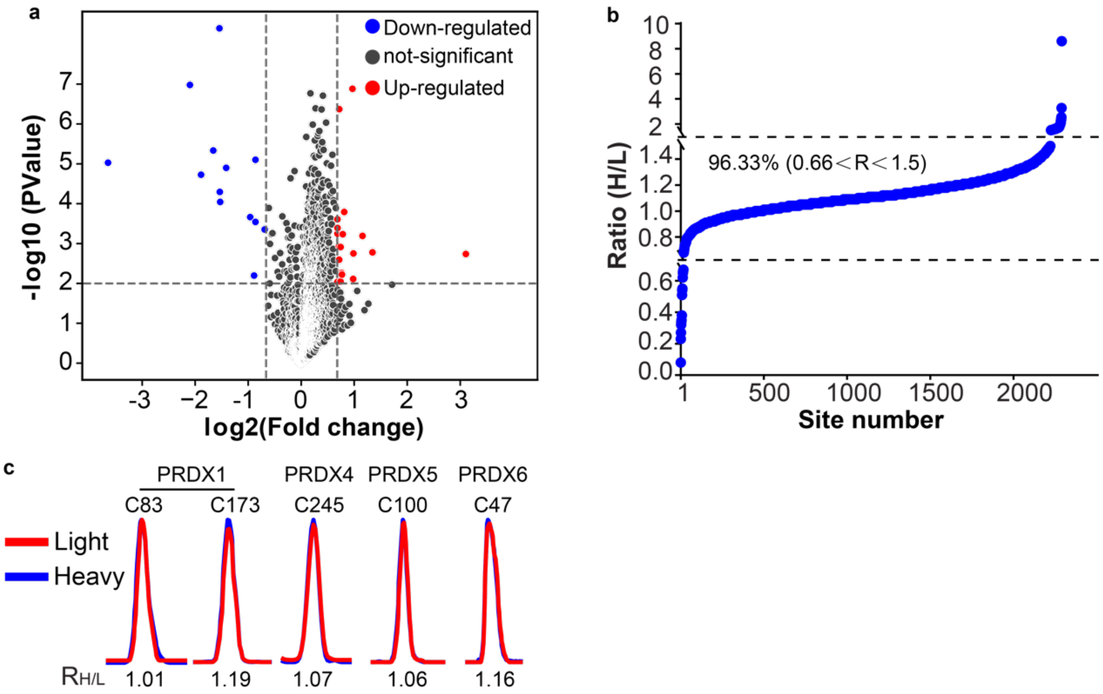

Supplementary Fig. 13 | *In situ* CysOx2 treatment does not perturb the cysteinome. HeLa cells were incubated with 0 or 10 μM CysOx2 at 37 °C for 1 h. Cell lysates were prepared and labeled with 100 μM thiol-reactive probe, IPM. The resulting IPM-modified peptides were conjugated to light (10 μM CysOx2) or heavy (0 μM, control) azido biotin reagents with photocleavable linker (Az-UV-biotin) via CuAAC. The light- and heavy-labeled samples were then mixed equally in amount and subjected to streptavidin-based enrichment. After washing, the modified peptides were selectively eluted from beads using 365 nm wavelength UV light for LC-MS/MS-based proteomic analysis. **a,** Volcano plot analysis showing that CysOx2 had no significant impact on the cysteinome (p-values≤0.05). **b,** Rank order of the determined $R_{H/L}$ values of IPM-sites with heavy (H) or light (L) tags from HeLa cells treated with or without CysOx2, respectively. **c,** Representative extracted-ion chromatograms from **b** further illustrating no change in IPM-tagged peptides from our well-known redox sensor proteins, including PRDX1 (C83, C173), PRDX4 (C245), PRDX5 (C100), and PRDX6 (C47) from HeLa cells treated with CysOx2. The profiles for light- and heavy-labeled peptides are shown in red and blue, respectively. Heavy (control) to light (treatment) ratios were calculated from biological duplicates and are displayed below the individual chromatograms.

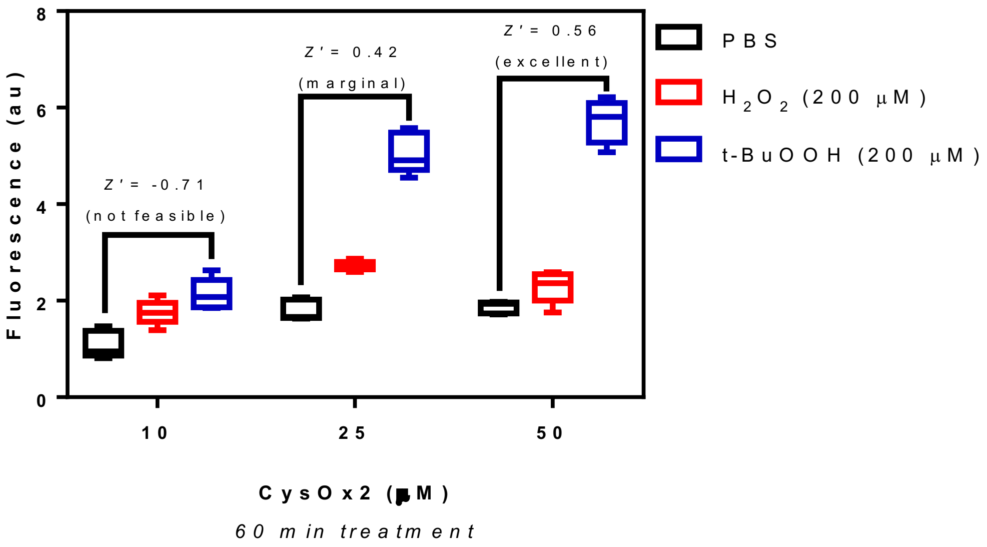

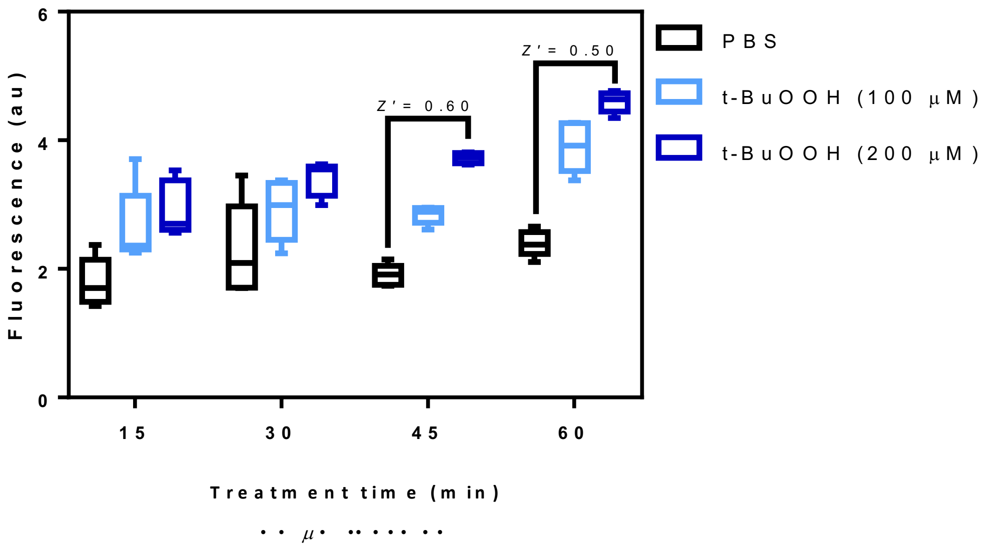

Supplementary Fig. 14 | Parameter optimization for the 96-well assay. Cells were treated with probe ± oxidant in DPBS containing 1% DMSO at 37 °C for 1 h. 50 µM CysOx2 and incubation times of 45-60 min produced significant difference between negative (PBS treated cells) and positive (oxidant treated cells) controls. Box plots show center line as median, whiskers show maxima and minima, and box limits show upper and lower quartiles. Data from *N* = 3 independent experiments and *N* = 5 independent readings per condition.

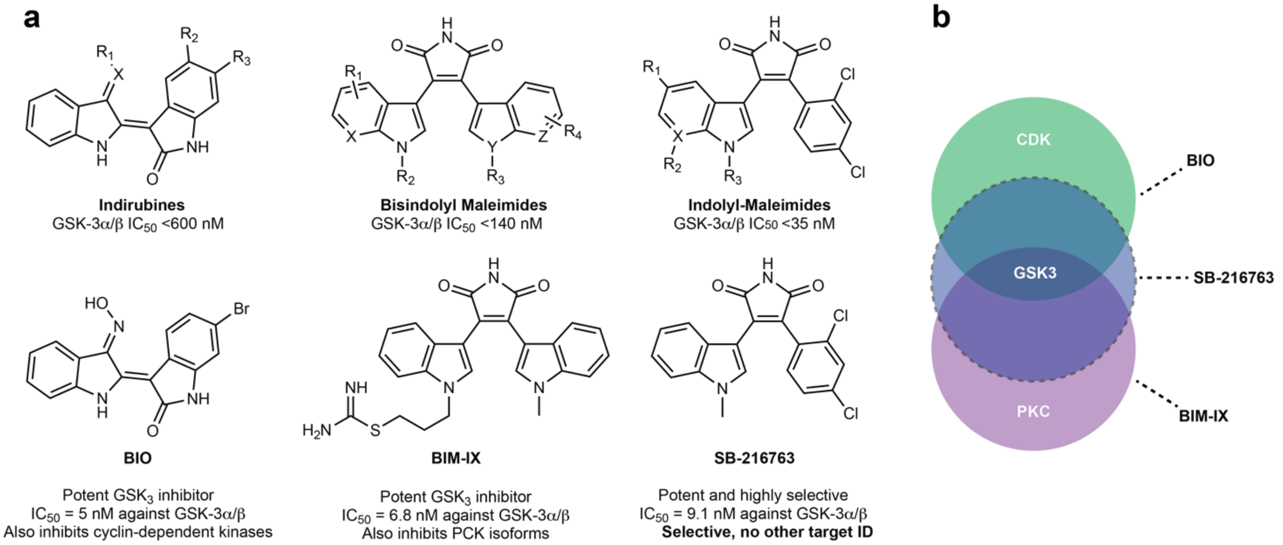

Supplementary Fig. 15 | GSK3 inhibitor potency and selectivity. **a,** GSK-3 inhibitor structures. **b,** Venn diagram conceptualizing the relationship between GSK3-specific inhibitor, SB-216763 (blue), and BIM-IX (purple; also inhibits PKC) and Bio (green; also inhibits cdks).

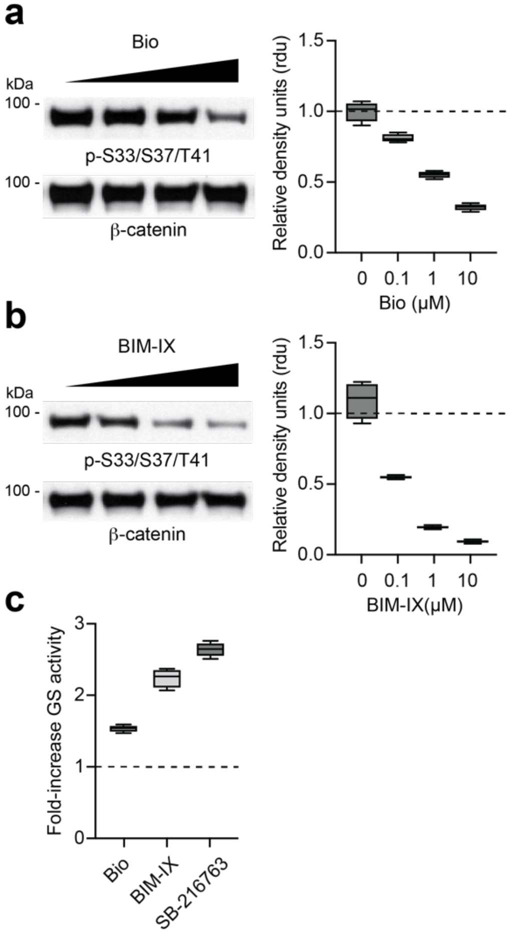

Supplementary Fig. 16. Effect of GSK3 inhibitors on β-catenin phosphorylation and glycogen synthase activity. **a-b,** Cells were treated with vehicle or with 0.1, 1 or 10 μM (left to right) SB-216763 for 30 min and then with 50 nM calyculin at 37 °C. Phospho-β-catenin-S33/S37/T41 and total β-catenin band densities were determined for all blots, expressed as relative density units (rdu). Box plots show center line as median, whiskers show maxima and minima, and box limits show upper and lower quartiles. *N* = *3* independent experiments. **c,** Serum-starved HeLa cells were treated for 60 min with 10 μM Bio, BIM-IX or SB-216763 or DMSO vehicle. Cells were harvested and lysates prepared. Lysate supernatants were assayed for GS activity as described in Supplementary Methods. Results are expressed as fold-increase in the GS activity ratio observed in control cells treated with DMSO vehicle. Error bars represent ±SEM. Box plots show center line as median, whiskers show maxima and minima, and box limits show upper and lower quartiles. *N* = 4 independent experiments.

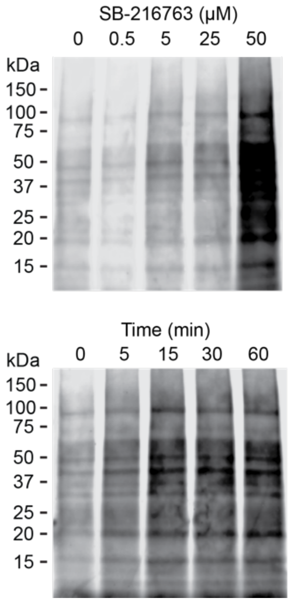

Supplementary Fig. 17 | In-gel detection of protein sulfenation of GSK3 inhibitor-treated HeLa cells. HeLa cells were treated with SB-216763 (0 – 50 µM) in presence of BTD (1 mM) in PBS containing 1% DMSO at 37 ºC for 1 h (top) or with SB-216763 (50 µM) and BTD (1 mM) for for 0 – 60 min (bottom). After cell lysis, lysates were clicked to TAMRA-azide and analyzed by SDS-PAGE. The fluorescence signal of the gels were measured in the 520 nm channel to detect TAMRA incorporation. *N = 2* independent experiments.

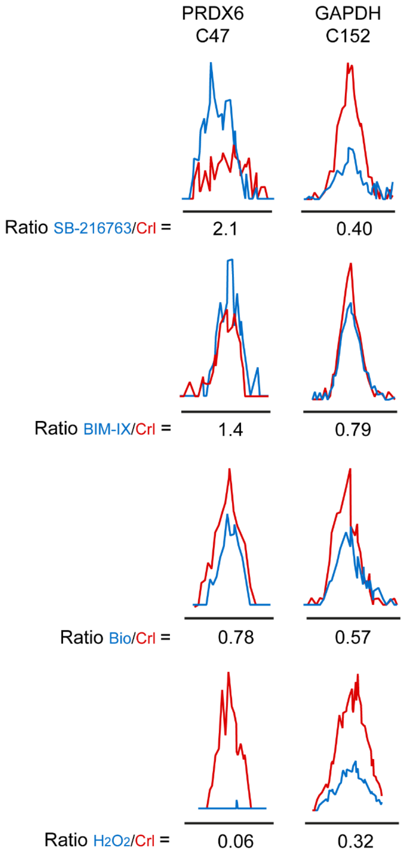

Supplementary Fig. 18 | Representative extracted ion chromatograms (XICs) showing changes in BTD-labeled peptides from PRDX6 and GAPDH. The profiles derived from inhibitor/H_2O_2 treated and control samples are shown in blue and red, respectively. The average R values calculated from two biological replicates are displayed below each individual XIC. Note: the XICs for the H_2O_2 treatment experiment were made using the RAW data from a previously published work[4].

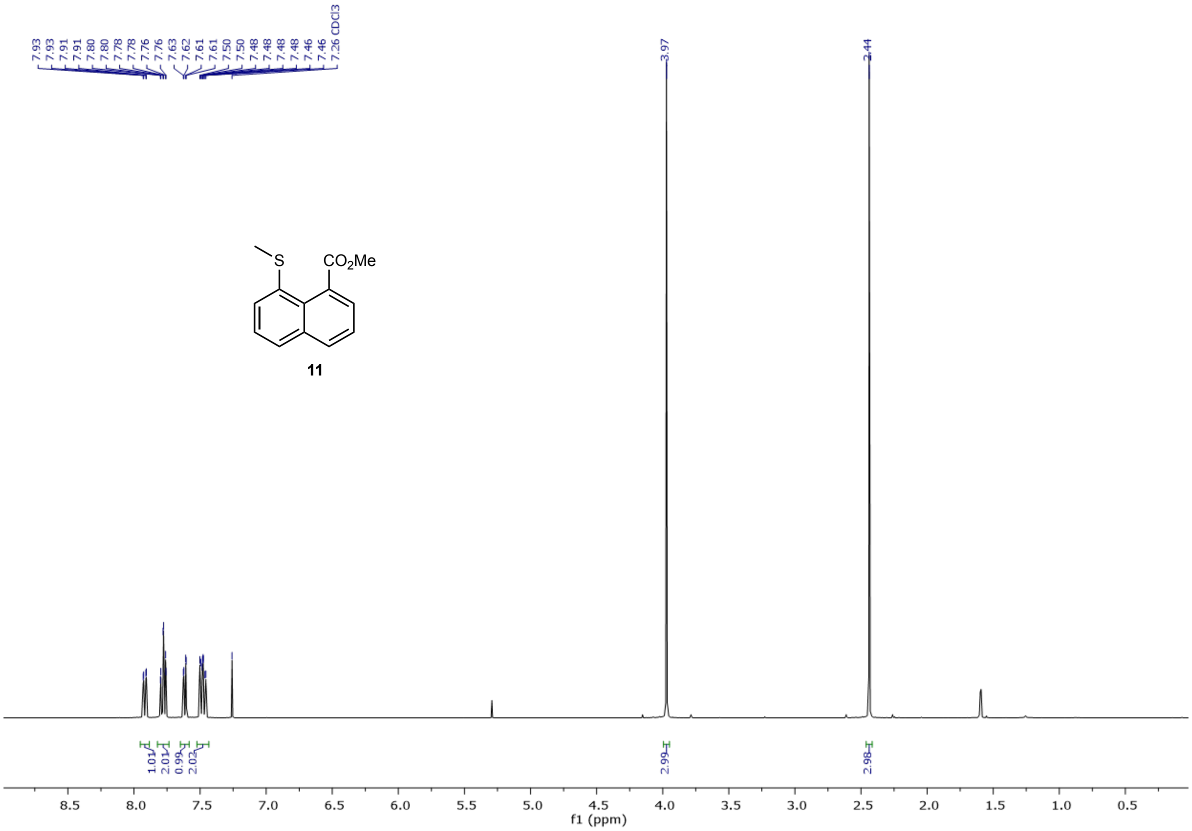
7.93
7.93
7.91
7.91
7.80
7.80
7.78
7.78
7.76
7.76
7.63
7.62
7.61
7.61
7.50
7.50
7.48
7.48
7.48
7.48
7.46
7.46
7.26 CDCl3
3.97
2.44
S
CO2Me
11
1.01
2.01
0.99
2.02
2.99
2.98
f1 (ppm)

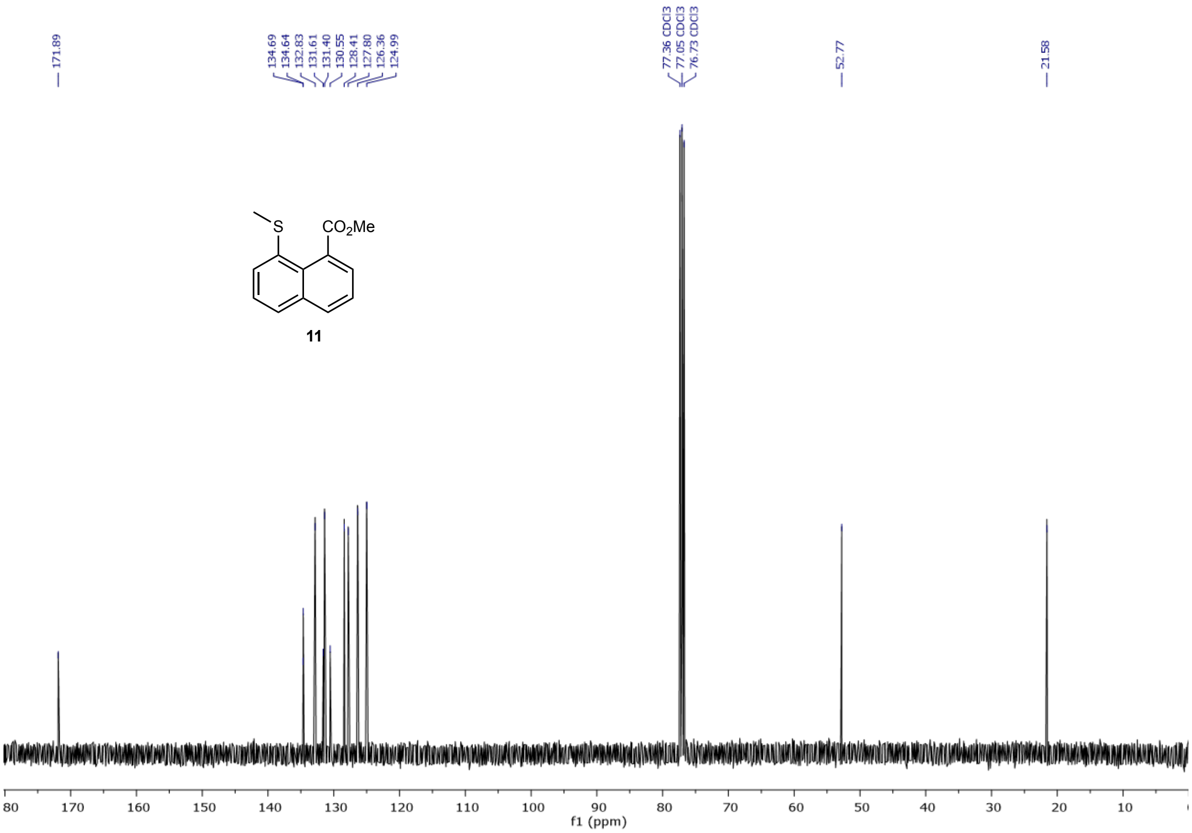
171.89
134.69
134.64
132.83
131.61
131.40
130.55
128.41
127.80
126.36
124.99
77.36 CDCl3
77.05 CDCl3
76.73 CDCl3
52.77
21.58
S
CO2Me
11
f1 (ppm)

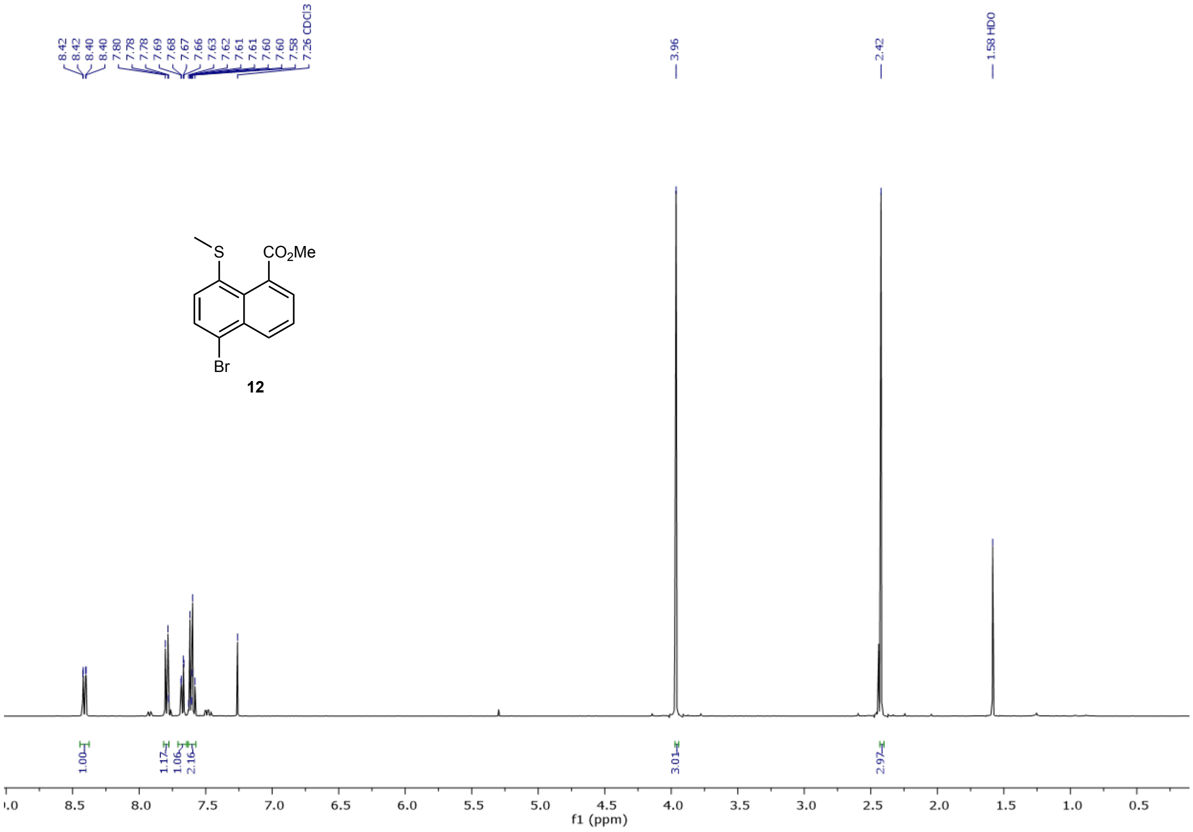
8.42 8.42 8.40 8.40 7.80 7.78 7.78 7.69 7.68 7.67 7.66 7.63 7.62 7.61 7.61 7.60 7.60 7.58 7.26 CDCl3
3.96
2.42
1.58 HDO
S
CO2Me
Br
12
1.00
1.17
1.06
2.16
3.01
2.97
f1 (ppm)

379

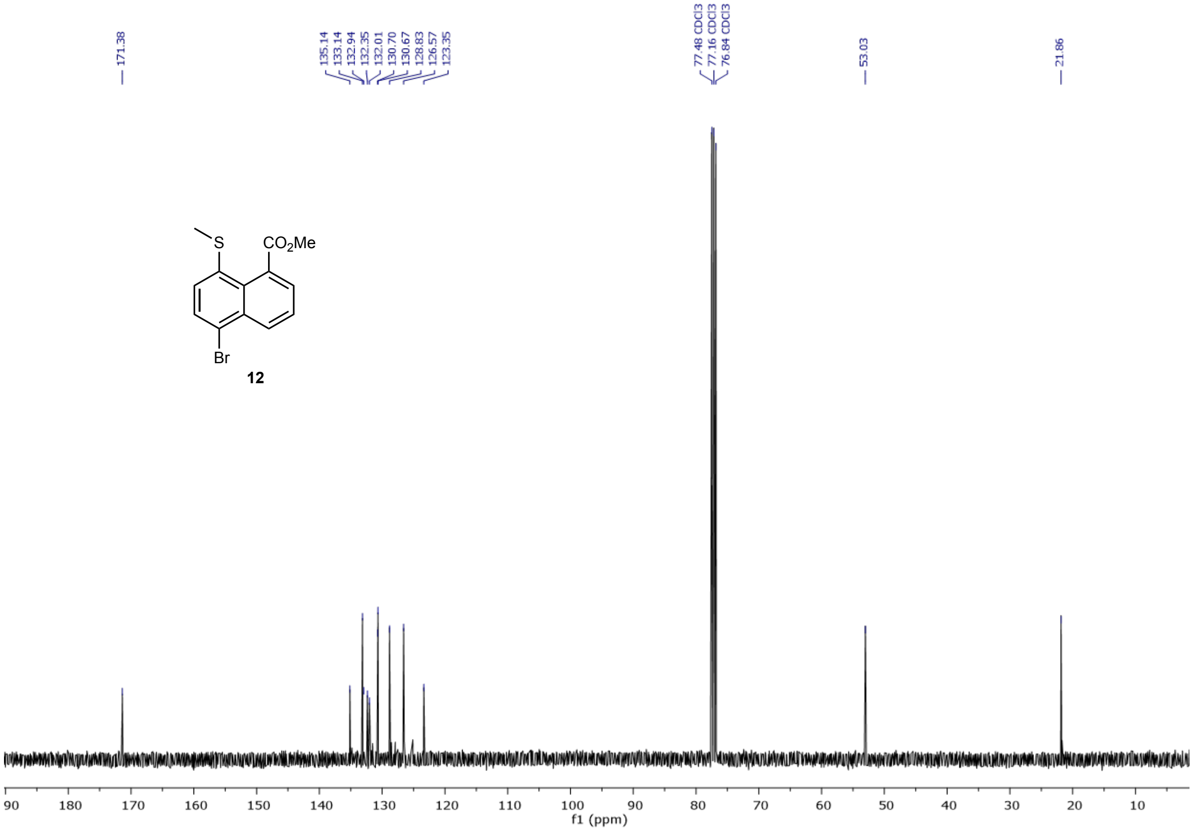
171.38
135.14 133.14 132.94 132.35 132.01 130.70 130.67 128.83 126.57 123.35
77.48 CDCl3 77.16 CDCl3 76.84 CDCl3
53.03
21.86
S
CO2Me
Br
12
f1 (ppm)

380

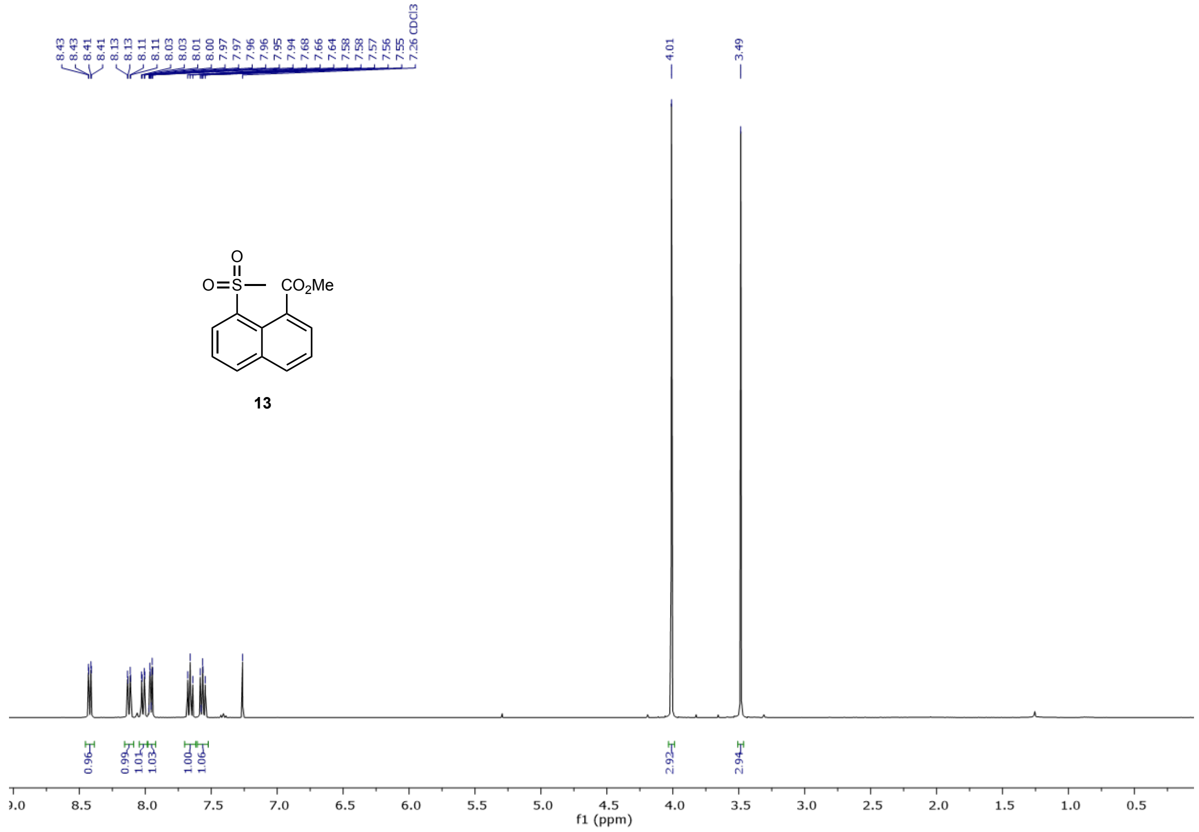
8.43
8.43
8.41
8.41
8.13
8.13
8.11
8.11
8.03
8.03
8.01
8.00
7.97
7.97
7.96
7.96
7.95
7.94
7.68
7.66
7.64
7.58
7.58
7.57
7.56
7.55
7.26 CDCl3
4.01
3.49
O
O
S
CO2Me
13
0.96
0.99
1.01
1.03
1.00
1.06
2.92
2.94
f1 (ppm)

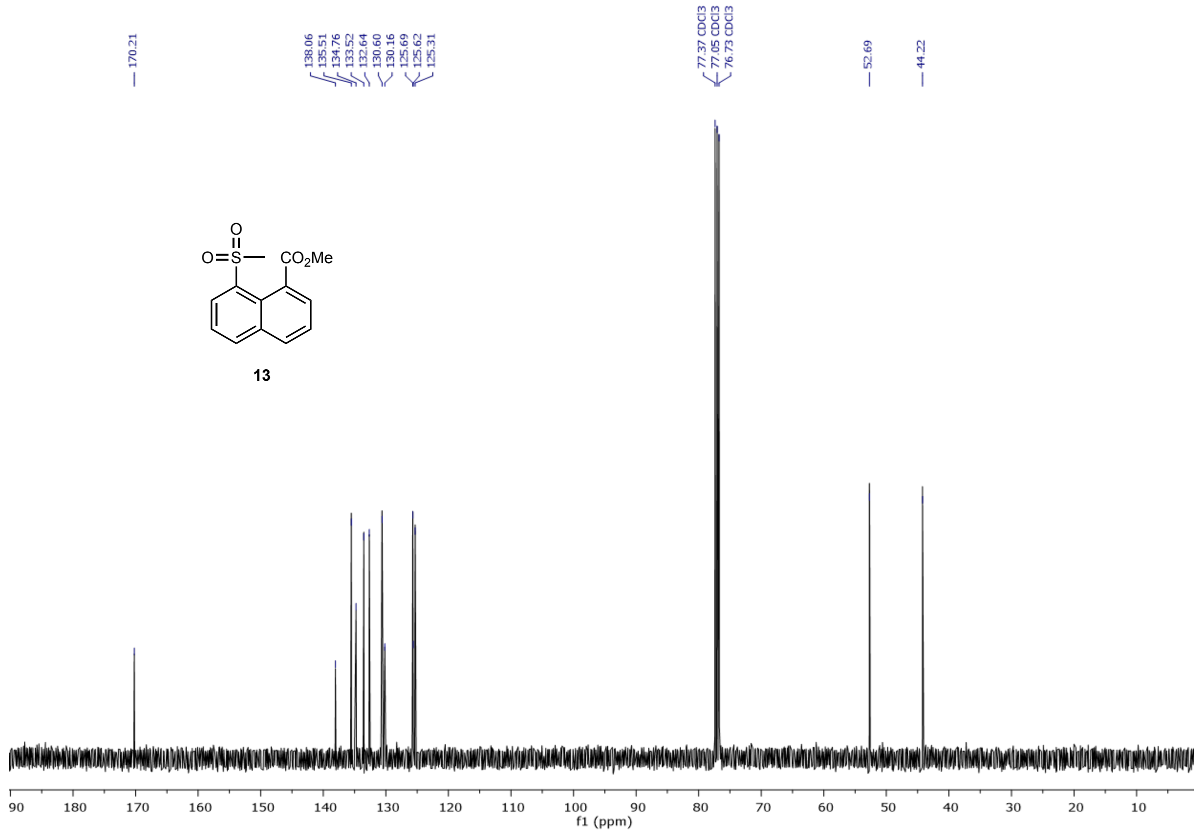
170.21
138.06
135.51
134.76
133.52
132.64
130.60
130.16
125.69
125.62
125.31
77.37 CDCl3
77.05 CDCl3
76.73 CDCl3
52.69
44.22
O
O
S
CO2Me
13
f1 (ppm)

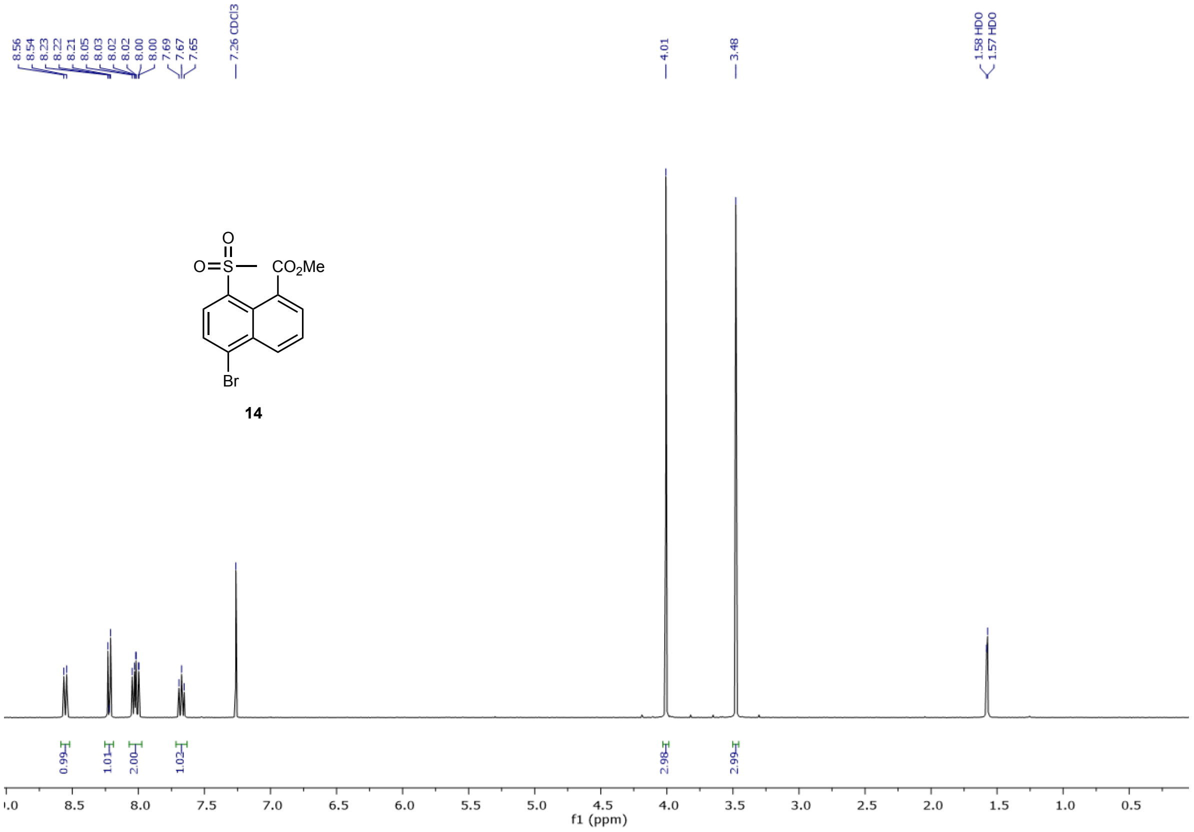
8.56 8.54 8.23 8.22 8.21 8.05 8.03 8.02 8.02 8.00 8.00 7.69 7.67 7.65
7.26 CDCl3
4.01
3.48
1.58 HDO 1.57 HDO
O=S CO2Me
Br
14
0.99 1.01 2.00 1.02 2.98 2.99
f1 (ppm)

383

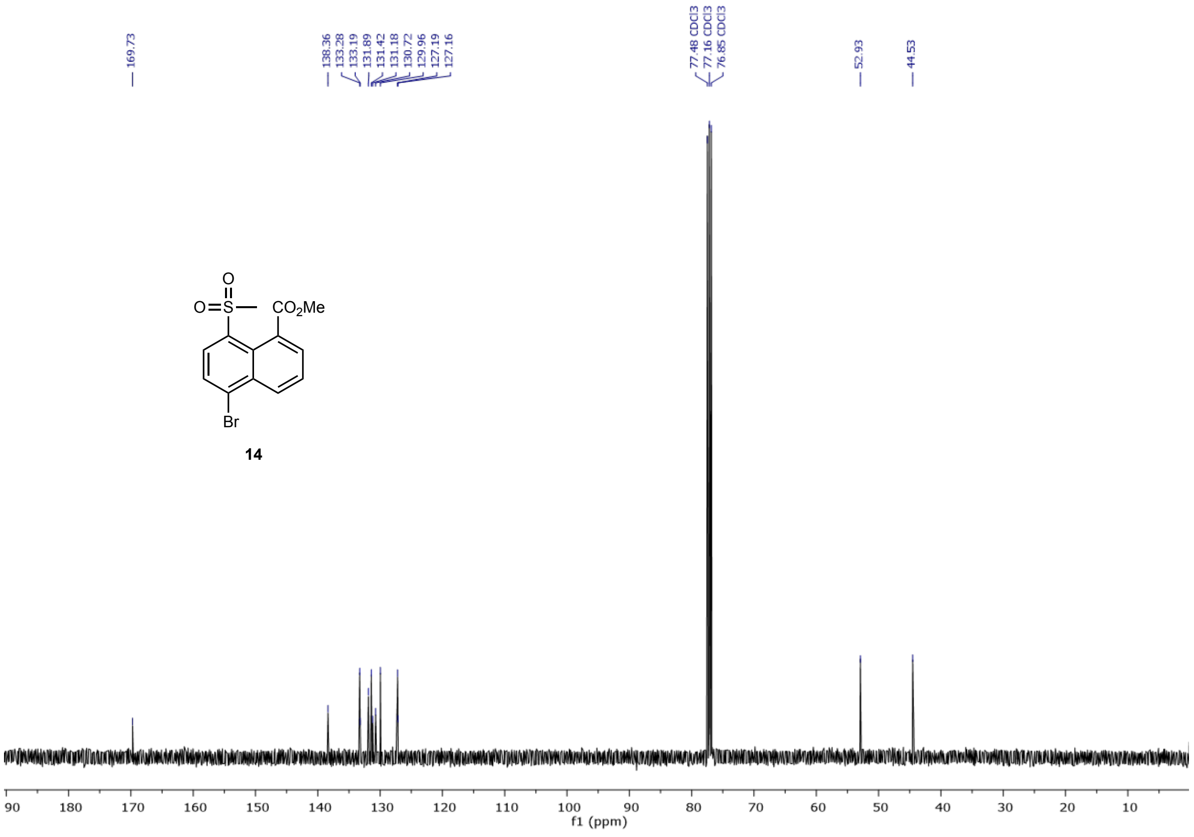
169.73
138.36 133.28 133.19 131.89 131.42 131.18 130.72 129.96 127.19 127.16
77.48 CDCl3 77.16 CDCl3 76.85 CDCl3
52.93
44.53
O=S CO2Me
Br
14
f1 (ppm)

384

385 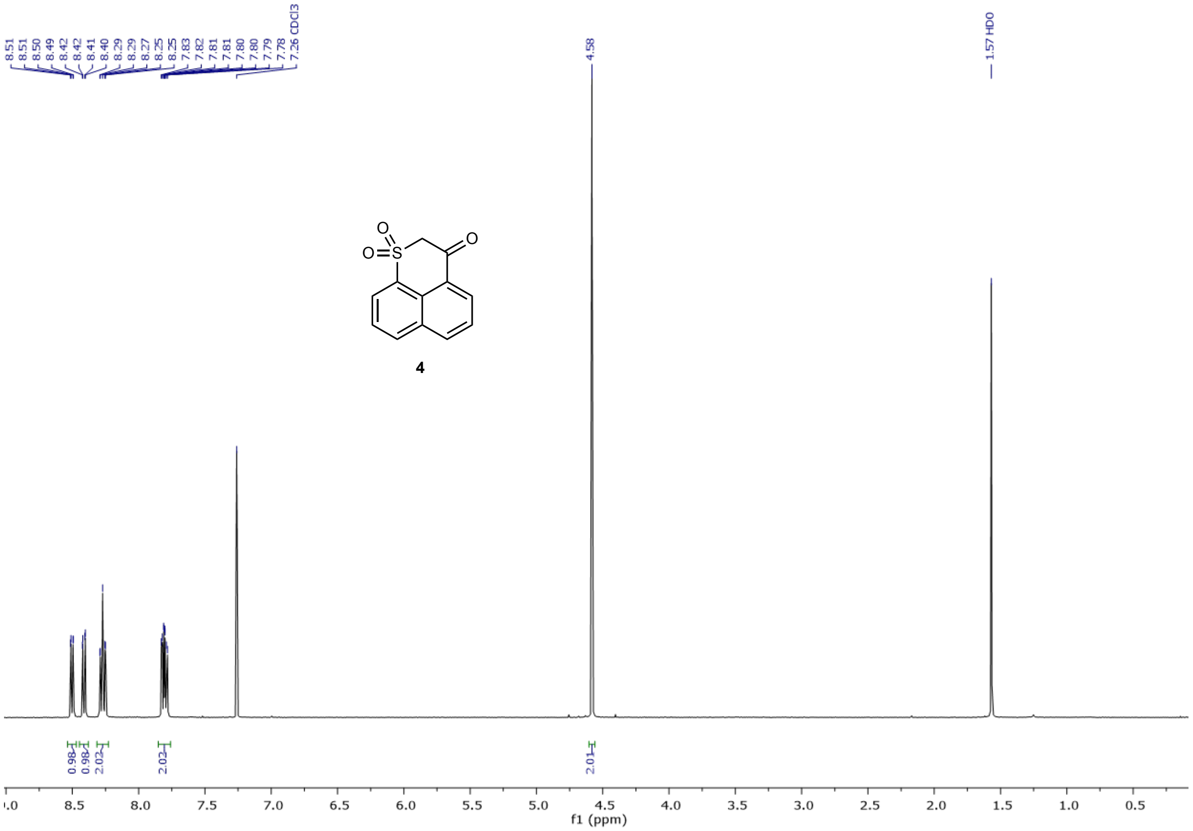
8.51
8.51
8.50
8.49
8.42
8.42
8.41
8.40
8.29
8.29
8.27
8.25
8.25
7.83
7.82
7.81
7.81
7.80
7.80
7.79
7.78
7.26 CDCl3
4.58
1.57 HDO
4
0.98
0.98
2.02
2.02
2.01
f1 (ppm)

386 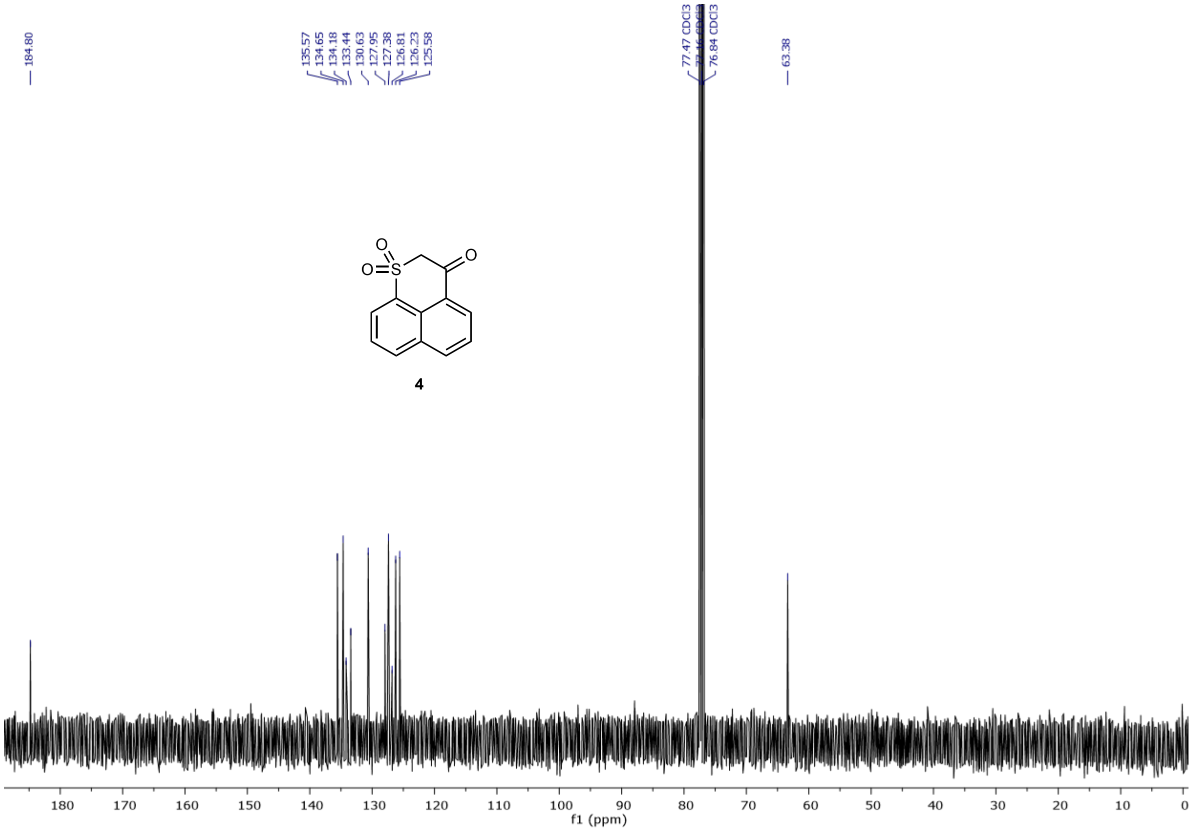
184.80
135.57
134.65
134.18
133.44
130.63
127.95
127.38
126.81
126.23
125.58
77.47 CDCl3
76.84 CDCl3
63.38
4
f1 (ppm)

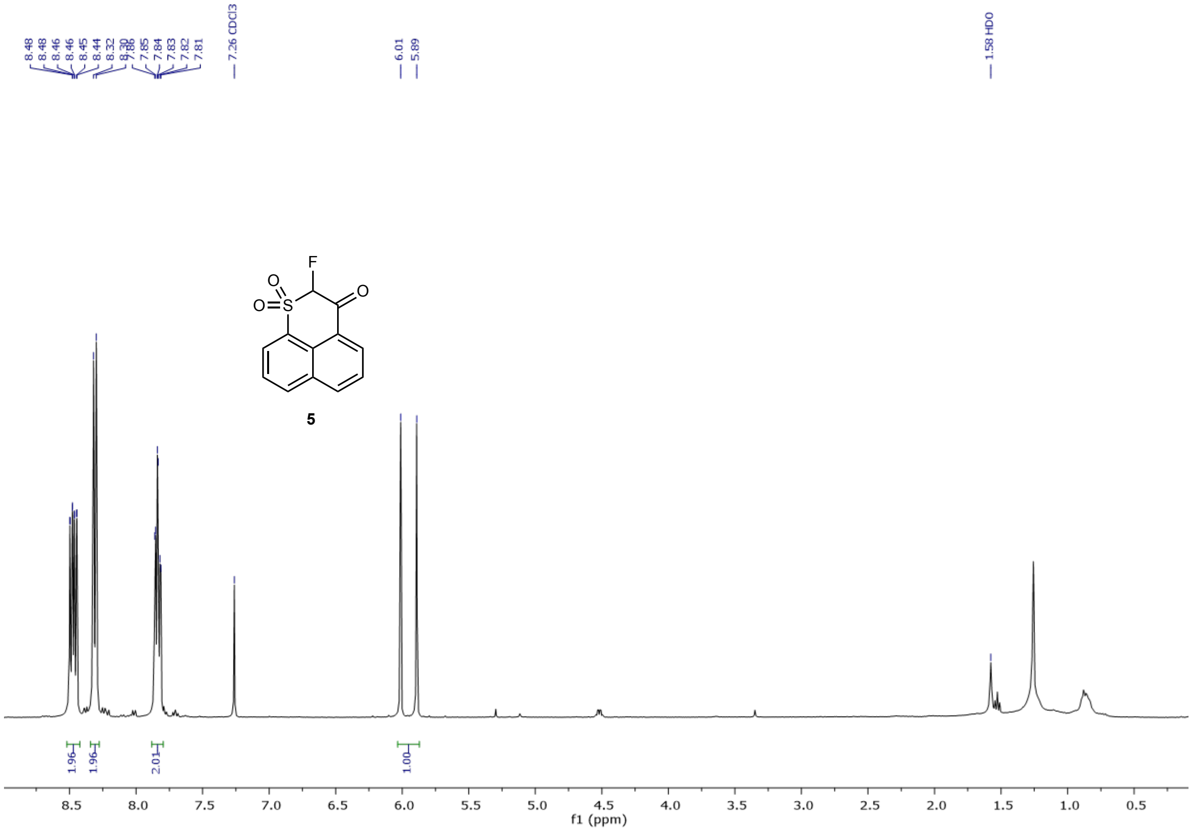
8.48
8.48
8.46
8.46
8.45
8.44
8.32
8.30
7.86
7.85
7.84
7.83
7.82
7.81
7.26 CDCl3
6.01
5.89
1.58 HDO
5
1.96
1.96
2.01
1.00
f1 (ppm)

387

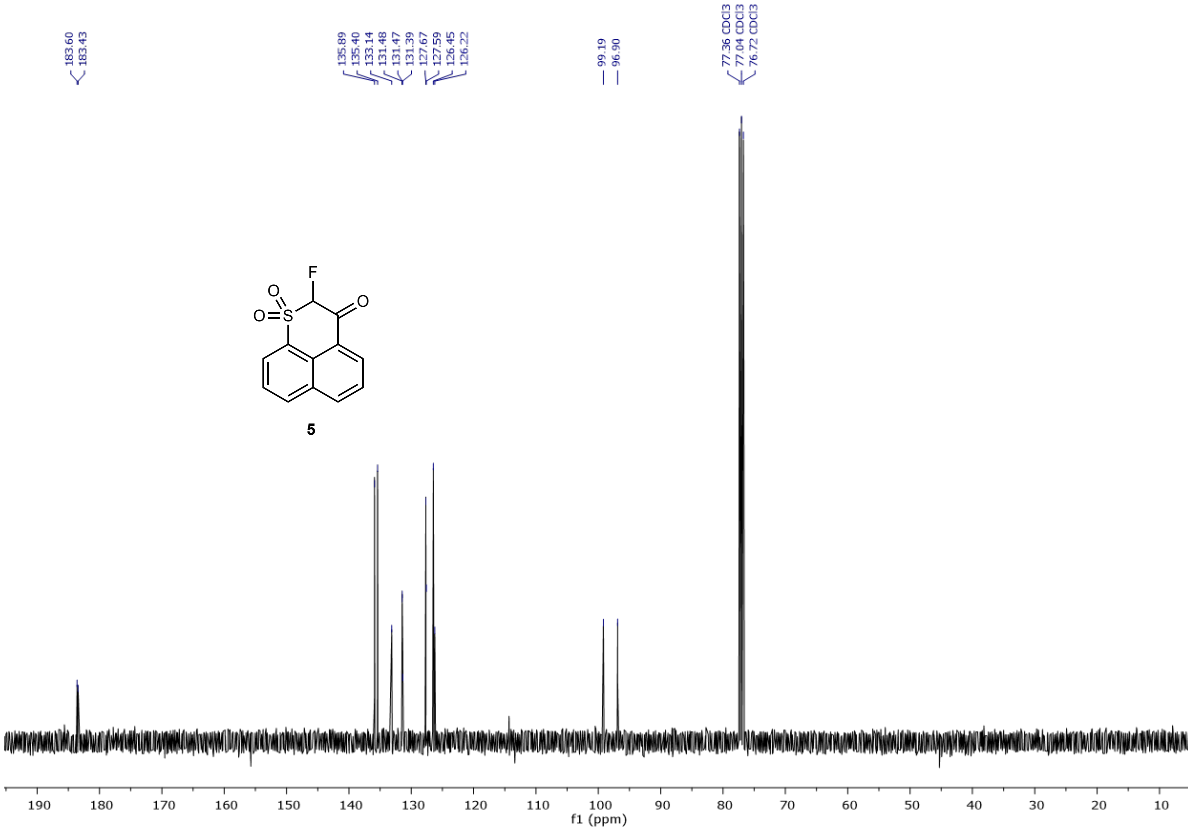
183.60
183.43
135.89
135.40
133.14
131.48
131.47
131.39
127.67
127.59
126.45
126.22
99.19
96.90
77.36 CDCl3
77.04 CDCl3
76.72 CDCl3
5
f1 (ppm)

388

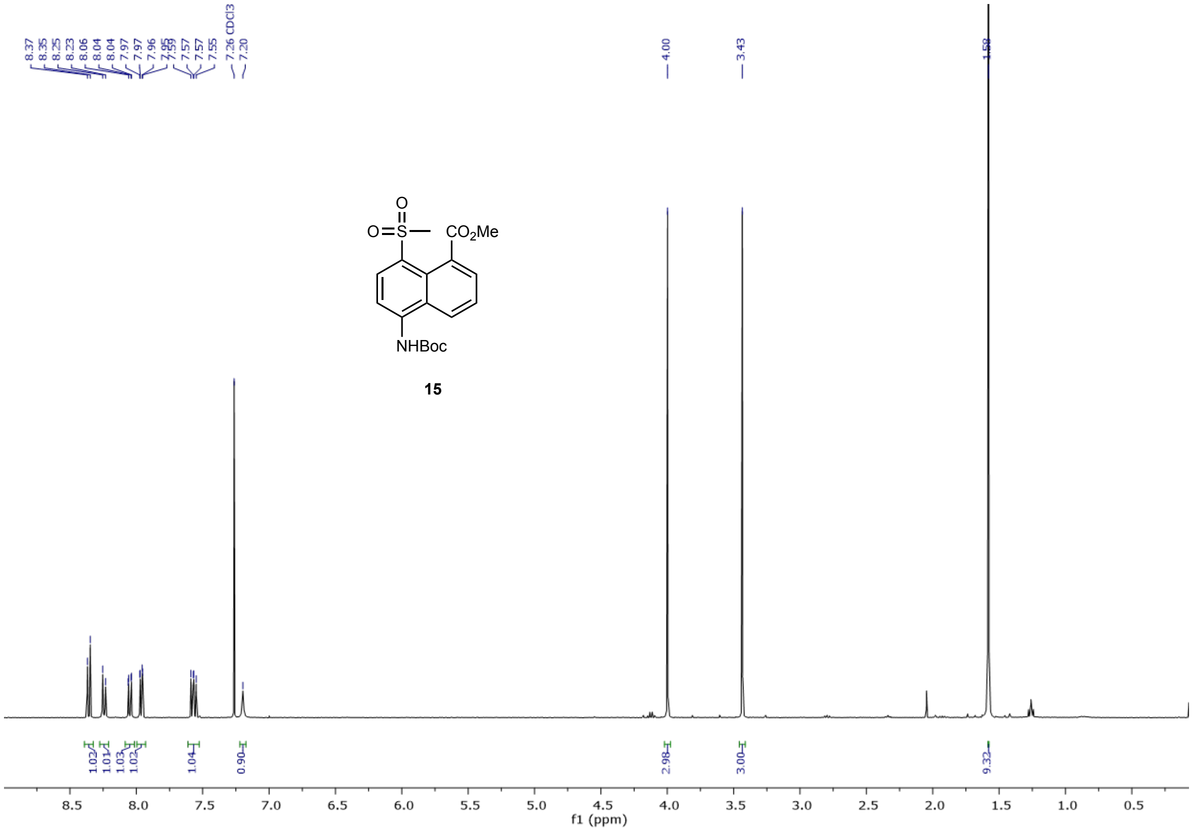
8.37
8.35
8.25
8.23
8.06
8.04
8.04
7.97
7.97
7.96
7.57
7.57
7.55
7.26 CDCl3
7.20
4.00
3.43
1.58
O
O=S—
CO2Me
NHBoc
15
1.02
1.01
1.03
1.02
1.04
0.90
2.98
3.00
9.32
f1 (ppm)

389

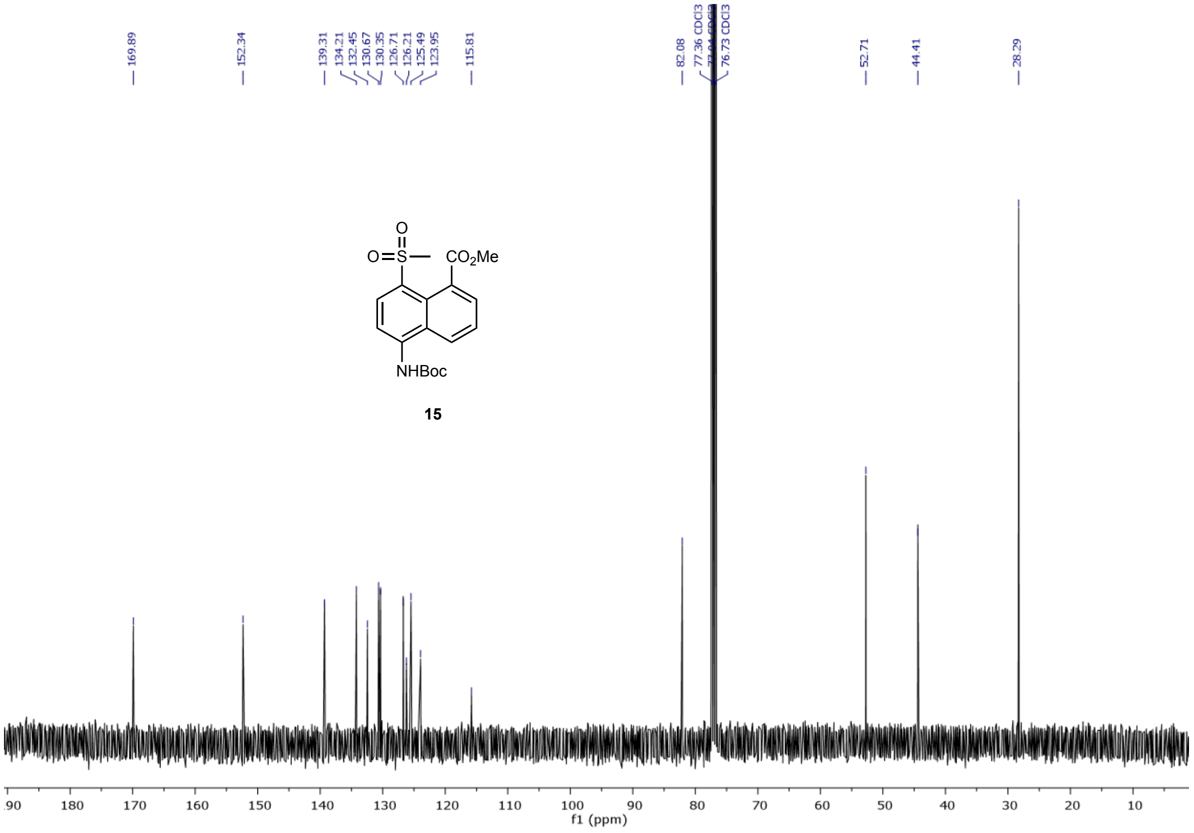
169.89
152.34
139.31
134.21
132.45
130.67
130.35
126.71
126.21
125.49
123.95
115.81
82.08
77.36 CDCl3
76.73 CDCl3
52.71
44.41
28.29
O
O=S—
CO2Me
NHBoc
15
f1 (ppm)

390

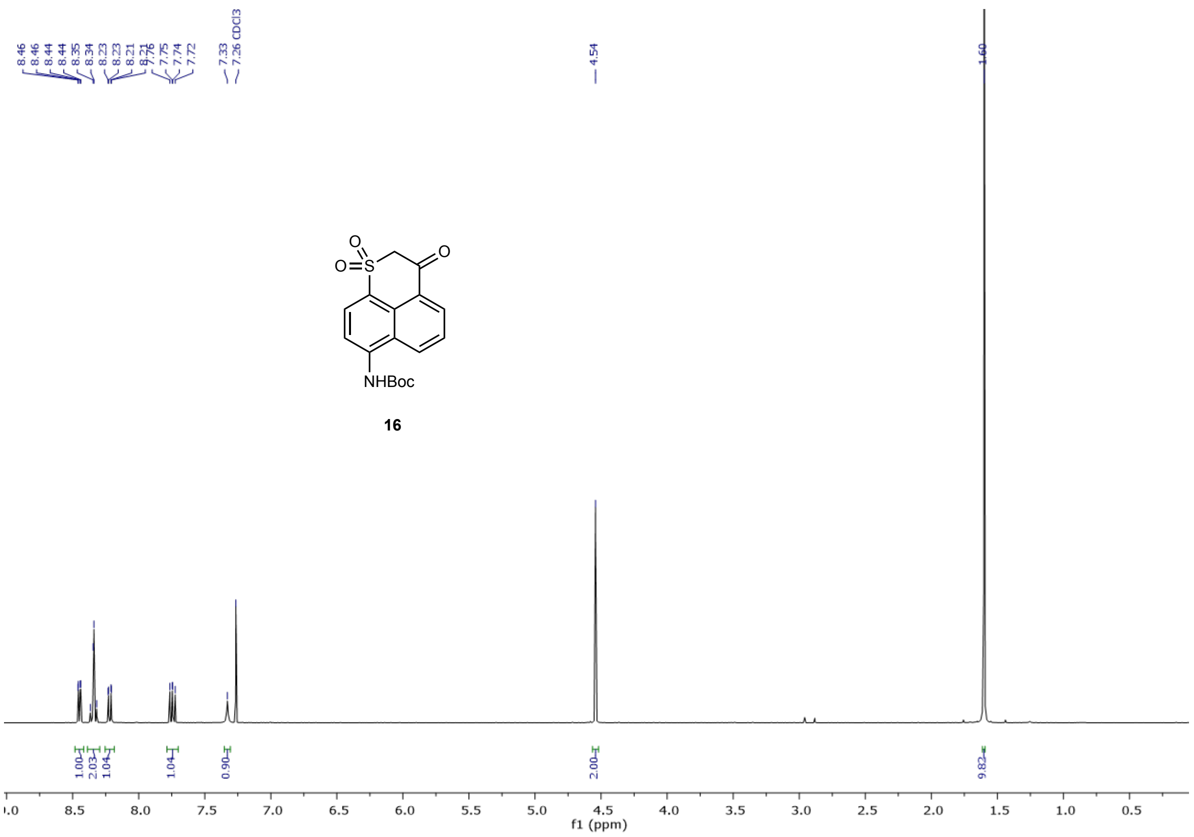
8.46
8.46
8.44
8.44
8.35
8.34
8.23
8.23
8.21
8.21
7.76
7.75
7.74
7.72
7.33
7.26 CDCl3
4.54
1.60
NHBoc
16
1.00
2.03
1.04
1.04
0.90
2.00
9.82
f1 (ppm)

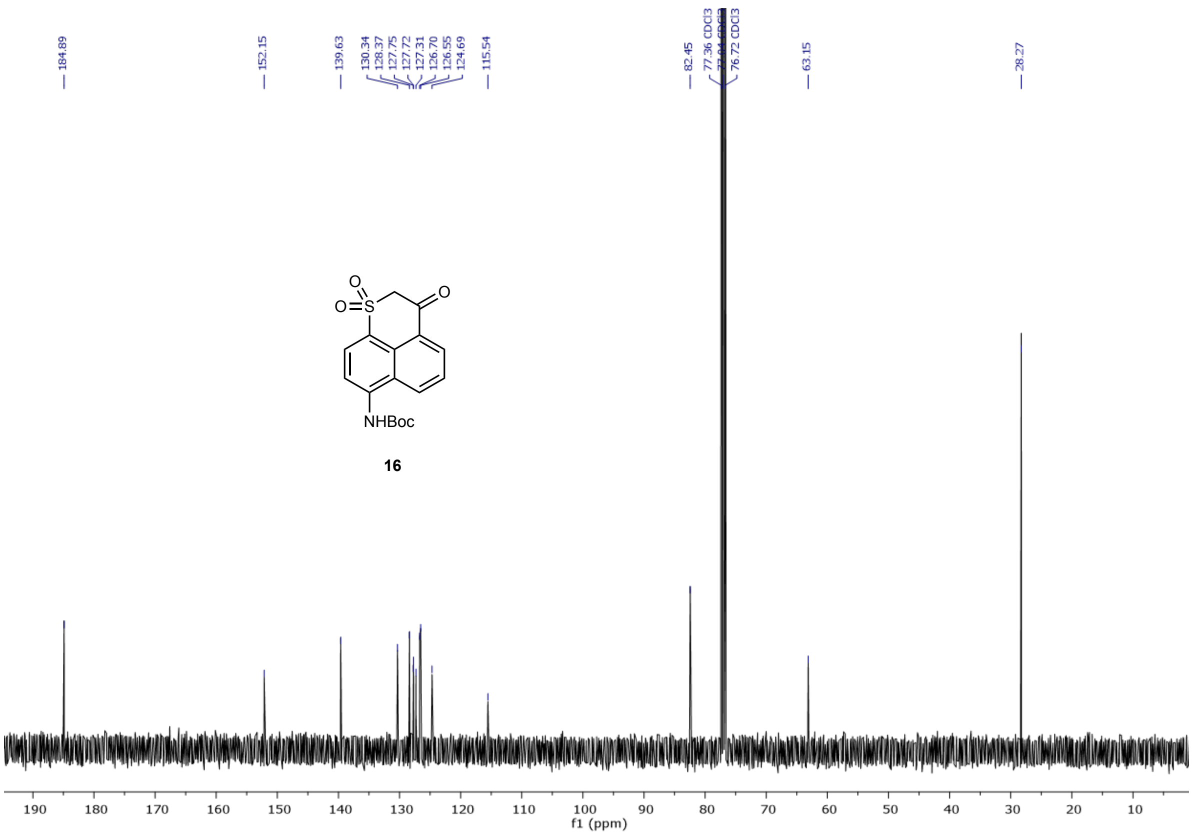
184.89
152.15
139.63
130.34
128.37
127.75
127.72
127.31
126.70
126.55
124.69
115.54
82.45
77.36 CDCl3
77.04 CDCl3
76.72 CDCl3
63.15
28.27
NHBoc
16
f1 (ppm)

393

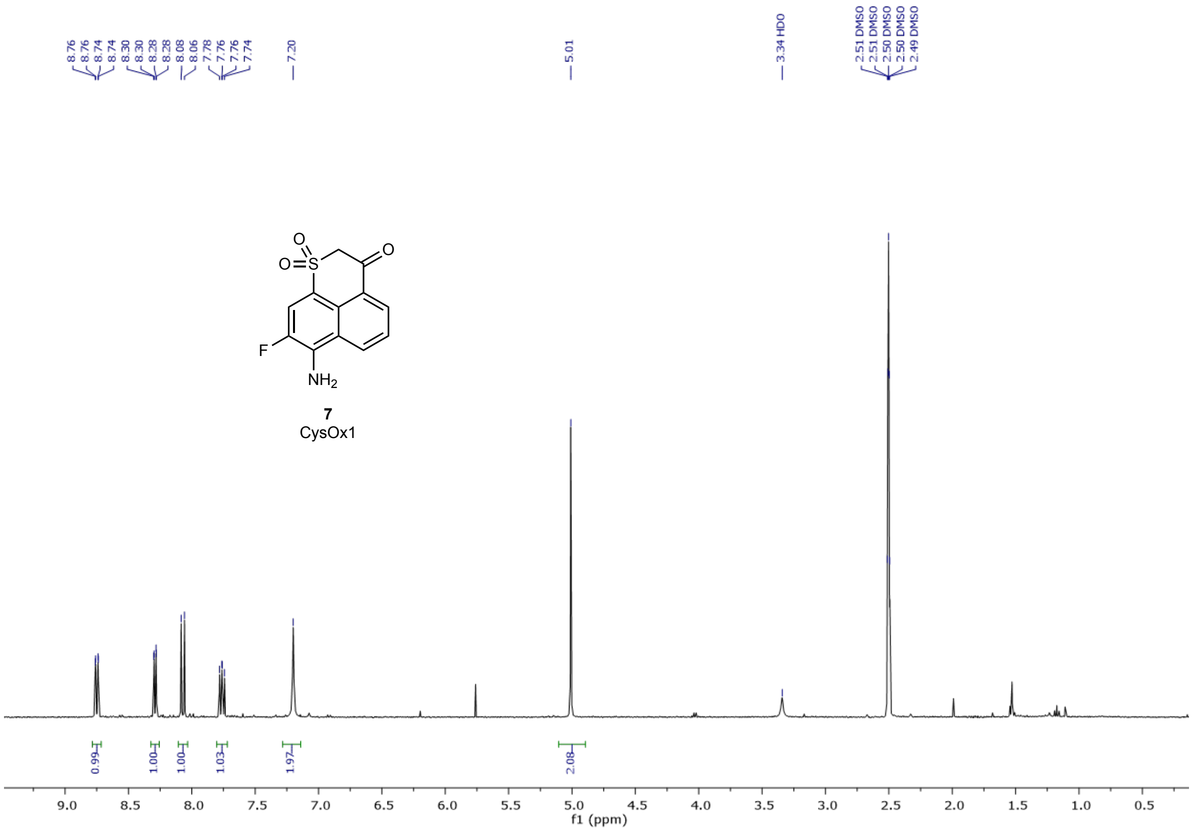

394

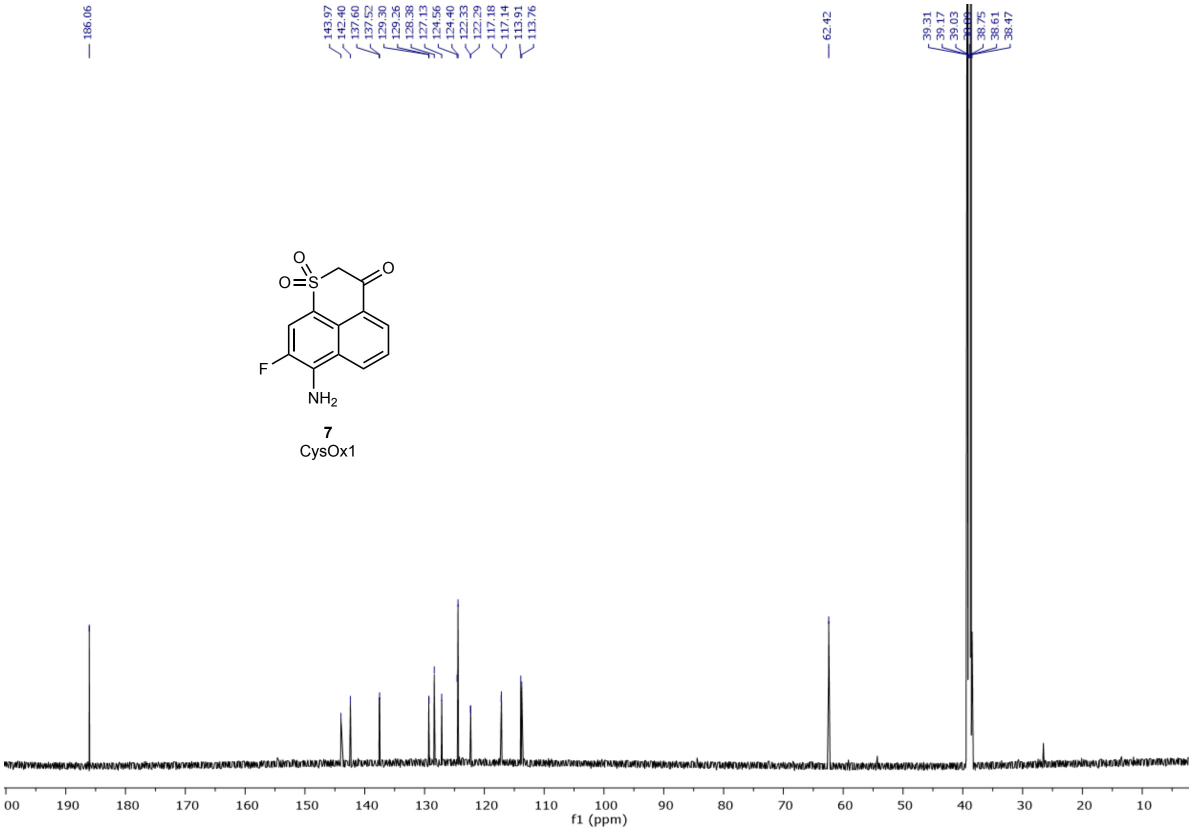

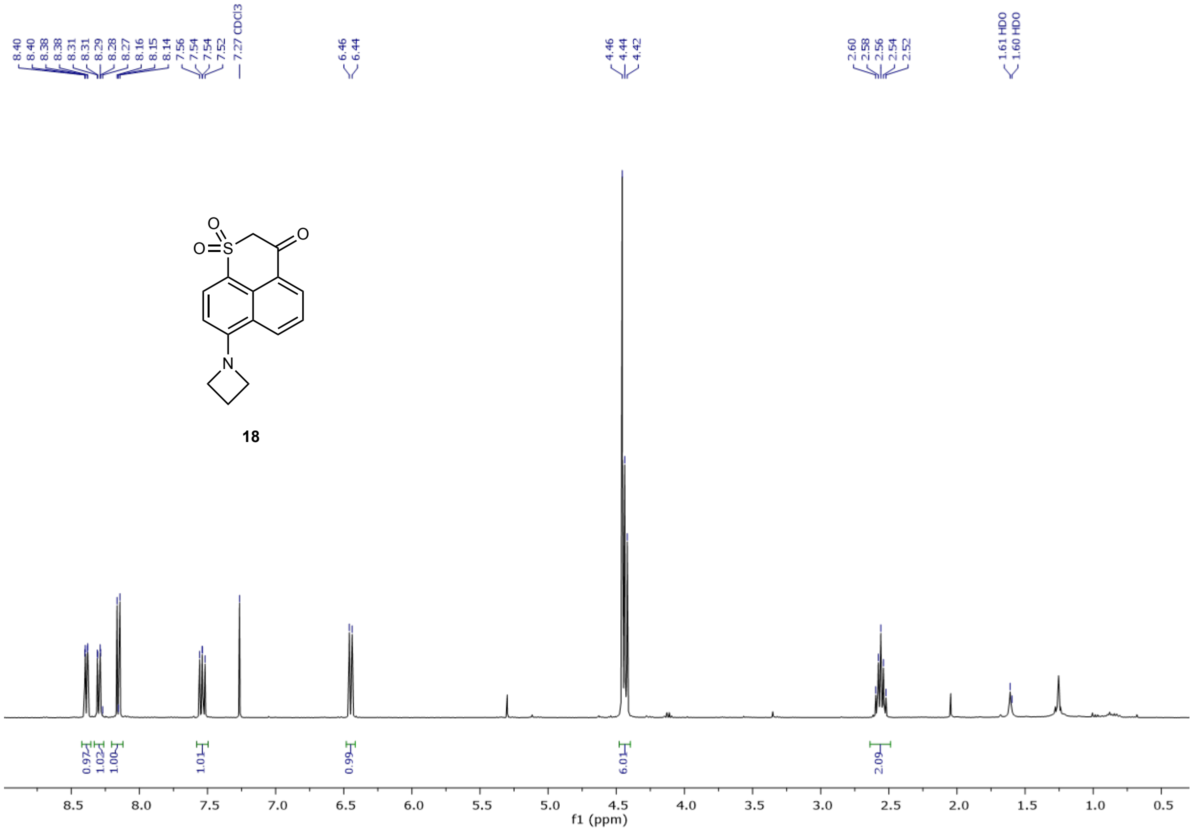
8.40
8.40
8.38
8.38
8.31
8.31
8.29
8.28
8.27
8.16
8.15
8.14
7.56
7.54
7.54
7.52
7.27 CDCl3
6.46
6.44
4.46
4.44
4.42
2.60
2.58
2.56
2.54
2.52
1.61 HDO
1.60 HDO
18
0.97
1.02
1.00
1.01
0.99
6.01
2.09
f1 (ppm)

395

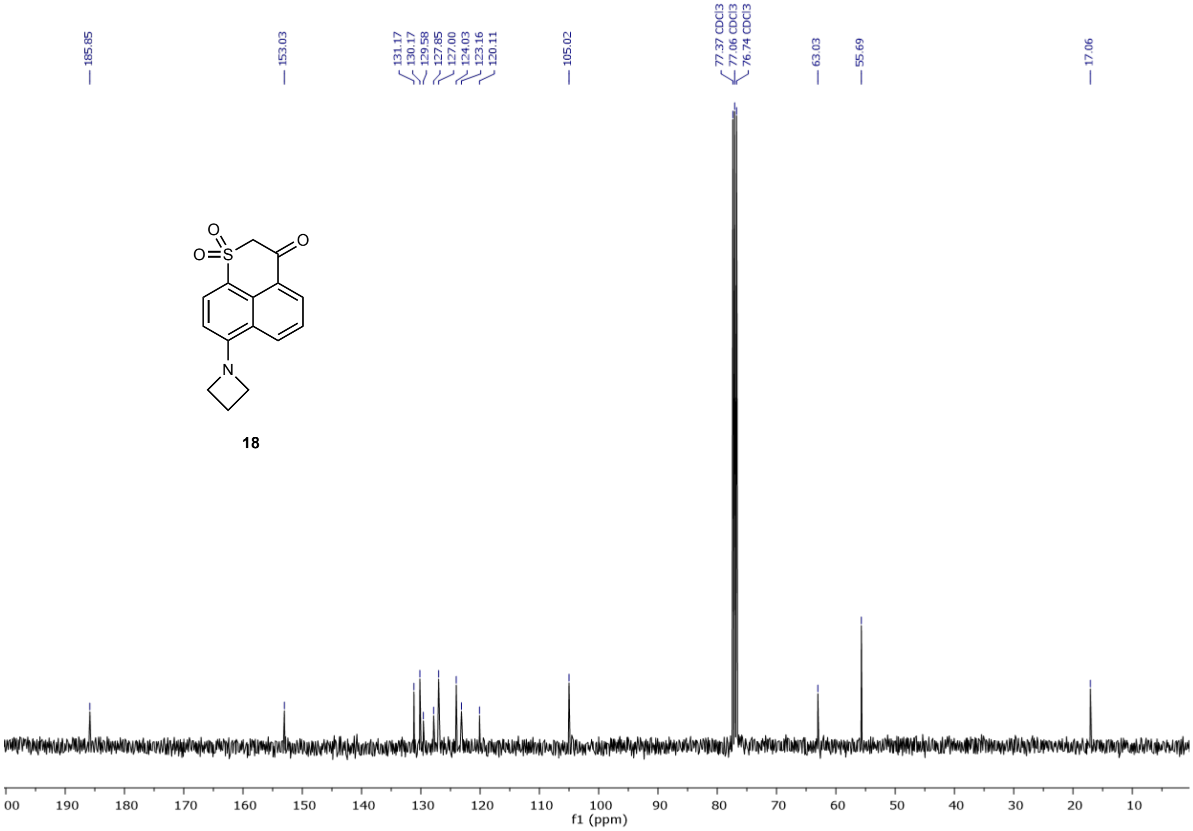
185.85
153.03
131.17
130.17
129.58
127.85
127.00
124.03
123.16
120.11
105.02
77.37 CDCl3
77.06 CDCl3
76.74 CDCl3
63.03
55.69
17.06
18
f1 (ppm)

396

397 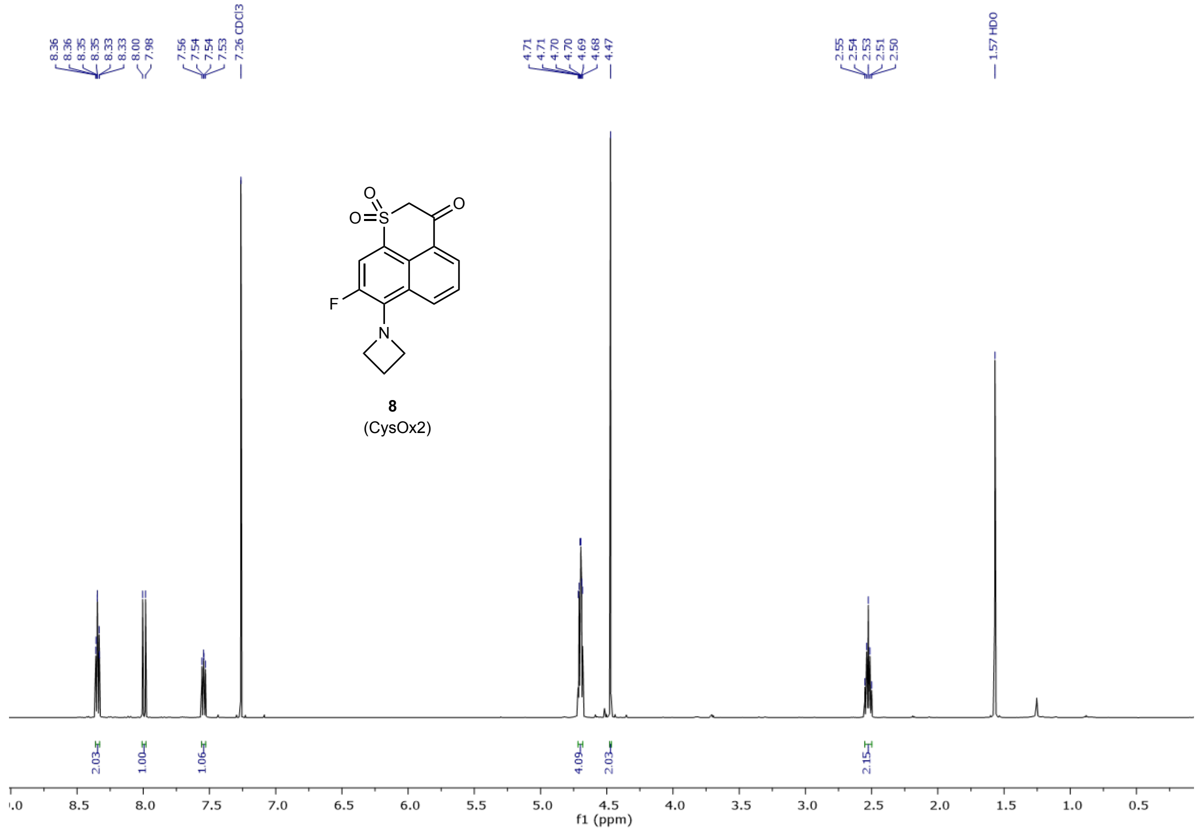
8.36
8.36
8.35
8.35
8.33
8.33
8.00
7.98
7.56
7.54
7.54
7.53
7.26 CDCl3
4.71
4.71
4.70
4.70
4.69
4.68
4.47
2.55
2.54
2.53
2.51
2.50
1.57 HDO
O
O
S
O
F
N
8
(CysOx2)
2.03
1.00
1.06
4.09
2.03
2.15
8.5
8.0
7.5
7.0
6.5
6.0
5.5
5.0
4.5
4.0
3.5
3.0
2.5
2.0
1.5
1.0
0.5
f1 (ppm)

398 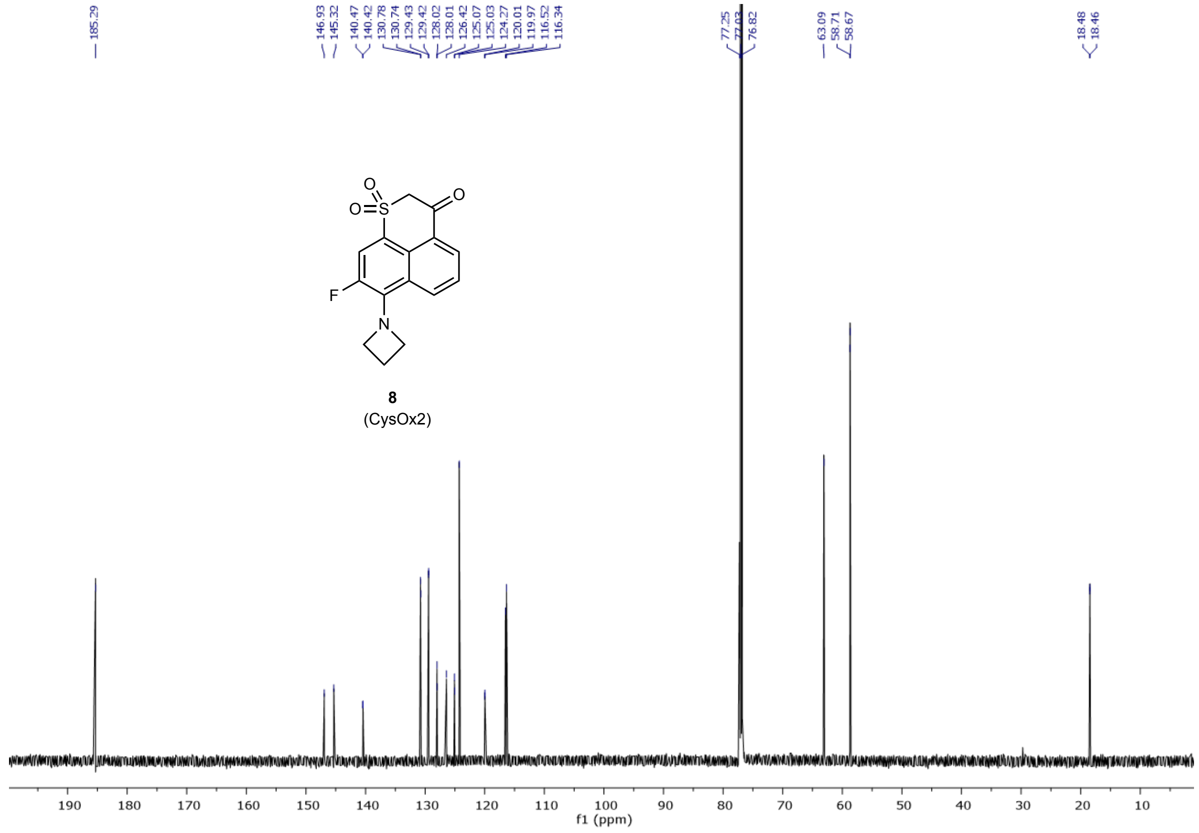
185.29
146.93
145.32
140.47
140.42
130.78
130.74
129.43
129.42
128.02
128.01
126.42
125.07
125.03
124.27
120.01
119.97
116.52
116.34
77.25
77.03
76.82
63.09
58.71
58.67
18.48
18.46
O
O
S
O
F
N
8
(CysOx2)
190
180
170
160
150
140
130
120
110
100
90
80
70
60
50
40
30
20
10
f1 (ppm)

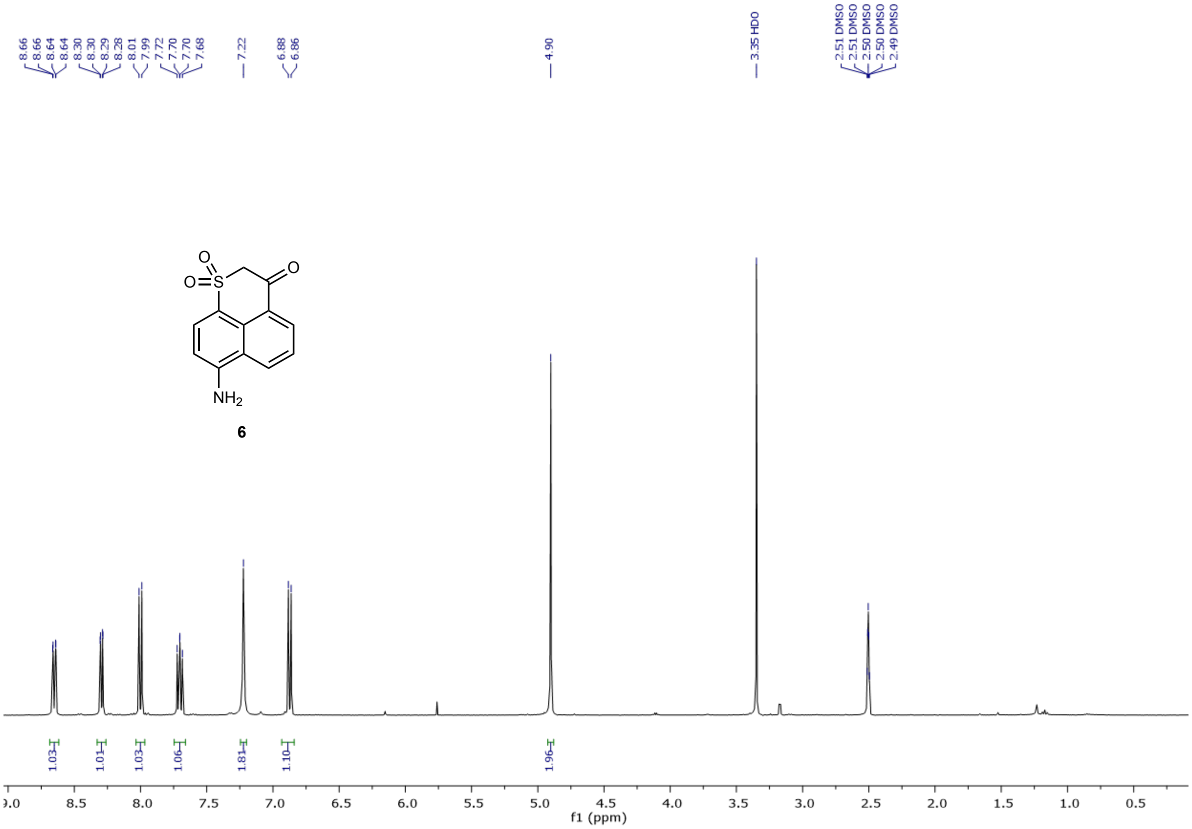
8.66
8.66
8.64
8.64
8.30
8.30
8.29
8.28
8.01
7.99
7.72
7.70
7.70
7.68
7.22
6.88
6.86
4.90
3.35 HDO
2.51 DMSO
2.51 DMSO
2.50 DMSO
2.50 DMSO
2.49 DMSO
O
O=S
O
NH2
6
1.03
1.01
1.03
1.06
1.81
1.10
1.96
8.5
8.0
7.5
7.0
6.5
6.0
5.5
5.0
4.5
4.0
3.5
3.0
2.5
2.0
1.5
1.0
0.5
f1 (ppm)

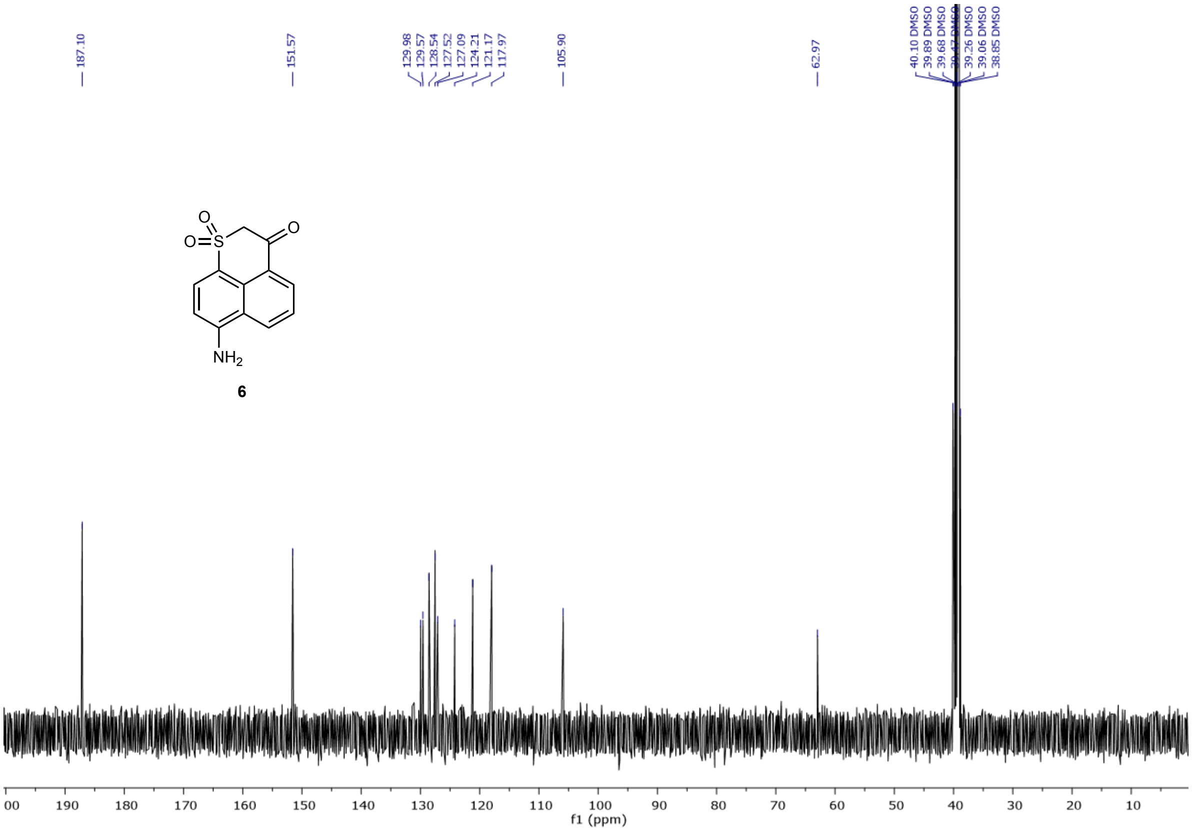
187.10
151.57
129.98
129.57
128.54
127.52
127.09
124.21
121.17
117.97
105.90
62.97
40.10 DMSO
39.89 DMSO
39.68 DMSO
39.26 DMSO
39.06 DMSO
38.85 DMSO
O
O=S
O
NH2
6
190
180
170
160
150
140
130
120
110
100
90
80
70
60
50
40
30
20
10
f1 (ppm)

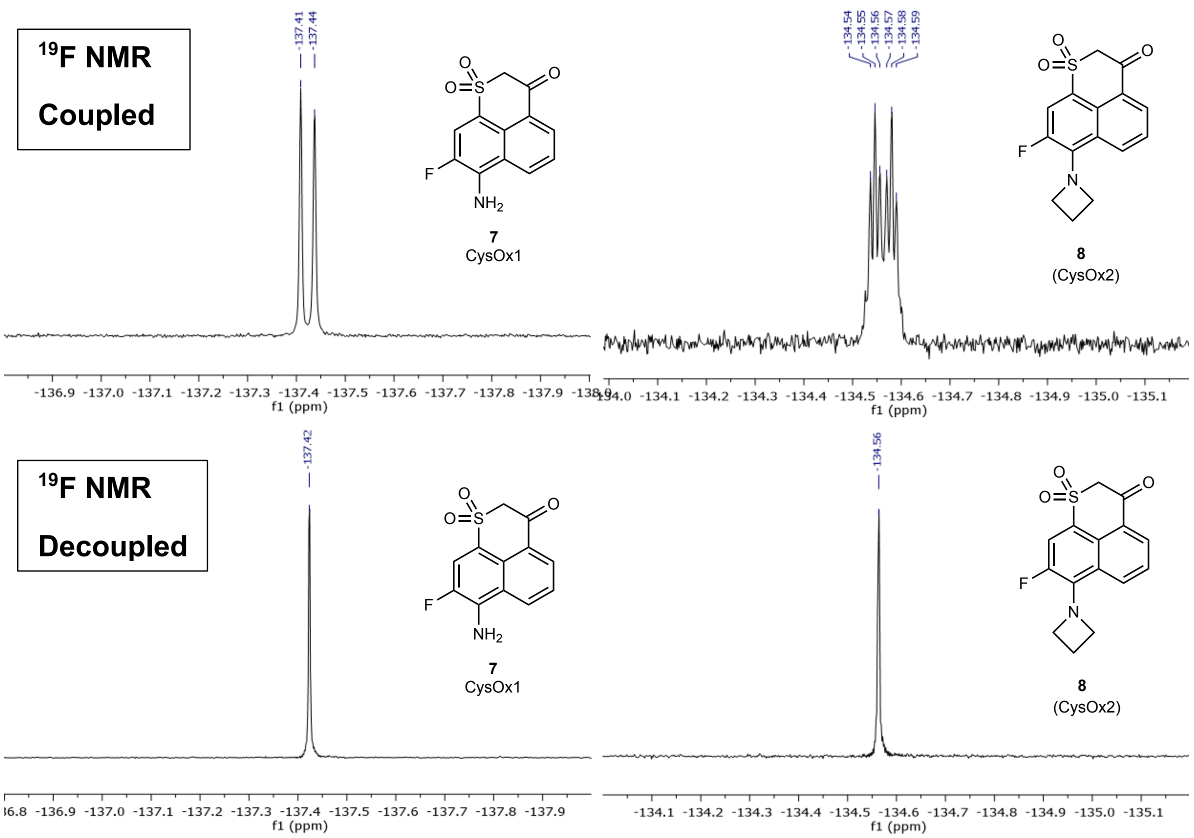

19F NMR
Coupled
-137.41
-137.44
7
CysOx1
-134.54
-134.55
-134.56
-134.57
-134.58
-134.59
8
(CysOx2)
f1 (ppm)
19F NMR
Decoupled
-137.42
7
CysOx1
-134.56
8
(CysOx2)
f1 (ppm)

REFERENCES

1. Gupta, V., Paritala, H. & Carroll, K. S. Reactivity, selectivity, and stability in sulfenic acid detection: A comparative study of nucleophilic and electrophilic probes. *Bioconjug. Chem.* **27**, 1411–1418 (2016).

2. Tom, C. T. *et al.* Chemoselective ratiometric imaging of protein S-sulfenation. *Chem. Commun.* **53**, 7385–7388 (2017).

3. Mitsudo, K. *et al.* Facile synthesis of naphthothiophenone derivatives and anthradithiophenedione *via* Friedel-Crafts acylation and their fundamental properties. *Synlett*. **27**, 2327–2332 (2016).
