# Supplementary material for: Reaction-based fluorogenic probes for detecting protein cysteine oxidation in living cells
Source: Nat Commun. 2022 Sep 21;13:5522. doi: 10.1038/s41467-022-33124-z (PMC9492777; doi:10.1038/s41467-022-33124-z)
Supplement: Supplementary file 3 — Reporting Summary [file 41467_2022_33124_MOESM3_ESM.pdf]

nature research

Corresponding author(s): Kate S. Carroll

Last updated by author(s): 8/31/2022

Reporting Summary

Nature Research wishes to improve the reproducibility of the work that we publish. This form provides structure for consistency and transparency in reporting. For further information on Nature Research policies, see our Editorial Policies and the Editorial Policy Checklist.

Statistics

For all statistical analyses, confirm that the following items are present in the figure legend, table legend, main text, or Methods section.

n/a	Confirmed	
☐	☒	The exact sample size (*n*) for each experimental group/condition, given as a discrete number and unit of measurement
☐	☒	A statement on whether measurements were taken from distinct samples or whether the same sample was measured repeatedly
☐	☒	The statistical test(s) used AND whether they are one- or two-sided<br>*Only common tests should be described solely by name; describe more complex techniques in the Methods section.*
☐	☒	A description of all covariates tested
☐	☒	A description of any assumptions or corrections, such as tests of normality and adjustment for multiple comparisons
☐	☒	A full description of the statistical parameters including central tendency (e.g. means) or other basic estimates (e.g. regression coefficient) AND variation (e.g. standard deviation) or associated estimates of uncertainty (e.g. confidence intervals)
☐	☒	For null hypothesis testing, the test statistic (e.g. F, t, r) with confidence intervals, effect sizes, degrees of freedom and P value noted<br>*Give P values as exact values whenever suitable.*
☒	☐	For Bayesian analysis, information on the choice of priors and Markov chain Monte Carlo settings
☒	☐	For hierarchical and complex designs, identification of the appropriate level for tests and full reporting of outcomes
☐	☒	Estimates of effect sizes (e.g. Cohen's *d*, Pearson's *r*), indicating how they were calculated

Our web collection on statistics for biologists contains articles on many of the points above.

Software and code

Policy information about availability of computer code

Data collection	Product formation of pseudo-first order reactions was monitored by a Agilent Technologies 1220 Infinity Liquid Chromatography system using the OpenLAB CDS vA.03.02.024 software. UV-Vis absorption spectra were recorded in a Varian Cary 300 Bio UV-Visible Spectrophotometer using the Cary WinUV v4.20 software. Fluorescence spectra was recorded in an Agilent Technologies Cary Eclipse Fluorescence Spectrophotometer using the Cary Eclipse v1.2 software. Fluorescence live-cell imaging was recorded in a Olympus FluoView IX81 confocal microscope using the FV10-ASW 3.0 software. Fluorescence measurements in 96-well plates were performed in a Molecular Devices SpectraMax M5 plate reader using the SoftMax Pro v5.4 software. LC-MS/MS Raw data files for proteomics studies were processed with the pFind studio (version 3.1.2, http://pfind.ict.ac.cn/software/pFind3/index.html) and pQuant (version 1.0) for peptide identification and quantification. Electrospray Ionization Mass Spectrometry (ESI-MS) of Gpx3 was measured on a Thermo Scientific LTQ XL linear ion trap mass spectrometer (Thermo Scientific) using MagTran software v1.0.
Data analysis	ImageJ v1.52a (https://imagej.nih.gov/ij/download.html) was used to image and analyze live cell imaging (Fig. 4a, S9a,b, S10, and S12). The deconvolution program MagTran was used to obtain the mass spectra of Gpx3 samples (Fig. 3a and S7a-c). Bar, line and box plots were were generated and analyzed by GraphPad Prism v9.4. Product formation traces of pseudo-first order reactions were generated and analyzed using Kaleidagraph v4.1.1 software.

For manuscripts utilizing custom algorithms or software that are central to the research but not yet described in published literature, software must be made available to editors and reviewers. We strongly encourage code deposition in a community repository (e.g. GitHub). See the Nature Research guidelines for submitting code & software for further information.

Data

Policy information about availability of data

All manuscripts must include a data availability statement. This statement should provide the following information, where applicable:

- Accession codes, unique identifiers, or web links for publicly available datasets
- A list of figures that have associated raw data
- A description of any restrictions on data availability

Data availability. The data supporting the findings from this study are available within the manuscript and its supplementary information. The chemoproteomic data sets have been deposited to the ProteomeXchange Consortium via the PRIDE partner repository with the dataset identifiers PXD029176. Source data are provided with this paper.

Field-specific reporting

Please select the one below that is the best fit for your research. If you are not sure, read the appropriate sections before making your selection.

[x] Life sciences [ ] Behavioural & social sciences [ ] Ecological, evolutionary & environmental sciences

For a reference copy of the document with all sections, see nature.com/documents/nr-reporting-summary-flat.pdf

Life sciences study design

All studies must disclose on these points even when the disclosure is negative.

Sample size	No statistical method was used to determine sample sizes. Sample sizes for imaging and cell-based assays were between 5 and 10 measurements per condition as stated in figure legends and were chosen at random.
Data exclusions	N/A
Replication	Experiments were replicated at least twice to guarantee reproducibility. The only exception was the initial kinase inhibitor screen, which was performed only once to identify potential hits for further evaluation. All attempts at replication of experiments were successful.
Randomization	N/A
Blinding	N/A

Reporting for specific materials, systems and methods

We require information from authors about some types of materials, experimental systems and methods used in many studies. Here, indicate whether each material, system or method listed is relevant to your study. If you are not sure if a list item applies to your research, read the appropriate section before selecting a response.

Materials & experimental systems

n/a	Involved in the study	
[ ]	[x]	Antibodies
[ ]	[x]	Eukaryotic cell lines
[x]	[ ]	Palaeontology and archaeology
[x]	[ ]	Animals and other organisms
[x]	[ ]	Human research participants
[x]	[ ]	Clinical data
[x]	[ ]	Dual use research of concern

Methods

n/a	Involved in the study	
[x]	[ ]	ChIP-seq
[x]	[ ]	Flow cytometry
[x]	[ ]	MRI-based neuroimaging

Antibodies

Antibodies used	*Describe all antibodies used in the study; as applicable, provide supplier name, catalog number, clone name, and lot number.*
Validation	*Describe the validation of each primary antibody for the species and application, noting any validation statements on the manufacturer's website, relevant citations, antibody profiles in online databases, or data provided in the manuscript.*

Eukaryotic cell lines

Policy information about cell lines

Cell line source(s)	HeLa cells were obtained from frozen stocks kept in a -130 C freezer, maintained by our lab.
Authentication	HeLa cells were authenticated by morphology.
Mycoplasma contamination	The cell lines were not tested for mycoplasma contamination.
Commonly misidentified lines (See ICLAC register)	N/A
