# Supplementary material for: Author Correction: Reaction-based fluorogenic probes for detecting protein cysteine oxidation in living cells
Source: Nat Commun. 2022 Nov 25;13:7274. doi: 10.1038/s41467-022-34953-8 (PMC9700698; doi:10.1038/s41467-022-34953-8)
Supplement: Supplementary file 1 — Description of Supplementary Files [file 41467_2022_34953_MOESM1_ESM.docx]

**Description of Supplementary Files**

For manuscript “Reaction-based fluorogenic probes for detecting protein cysteine oxidation in living cells”

**Supplementary Data 1.** Global profiling of cysteinome in the human cells treated with CysOX2.

**Supplementary Data 2.** Relative fluorescence intensity of HeLa cells treated with kinase inhibitors.

**Supplementary Data 3.** Proteome-wide mapping sulfenic acid ligandability in the human proteome.
